# Proton Transfer Equilibrium in Pseudoprotic Ionic Liquids: Inferences on Ionic Populations

**DOI:** 10.1021/acs.jpcb.4c07150

**Published:** 2025-01-17

**Authors:** Mark N. Kobrak, Dmytro Nykypanchuk, Ankit Jain, Eddie Louz, Andrzej A. Jarzecki

**Affiliations:** † Department of Chemistry and Biochemistry, Brooklyn College of the City University of New York, 2900 Bedford Ave., Brooklyn, New York 11210, United States; ‡ Department of Chemistry, The Graduate Center of the City University of New York, 365 Fifth Ave., New York, New York 10016, United States; § Center for Functional Nanomaterials, Brookhaven National Laboratory, Upton, New York 11973, United States; ∥ Department of Biochemistry, The Graduate Center of the City University of New York, 365 Fifth Ave., New York, New York 10016, United States

## Abstract

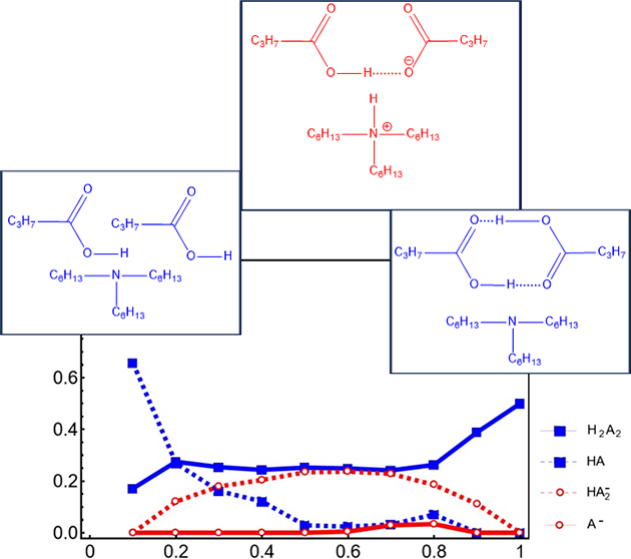

Nonstoichiometric pseudoprotic ionic liquids (NPPILs) are an emerging class of ionic liquids with interesting physical properties and intriguing prospects for technological applications. However, fundamental questions remain about the proton transfer equilibria that underlie their ionic character. We use a combination of nuclear magnetic resonance spectroscopy, infrared spectroscopy, and small-angle X-ray scattering to characterize the equilibria of trihexylamine/butyric acid and water/butyric acid mixtures. This combination of techniques offers considerable insight into how proton transfer changes with the composition of the mixture. Further, we construct a model based on information from ^1^H and ^13^C NMR, which yields numerical values for the concentrations of all ions present in the trihexylamine/butyric acid mixture, and demonstrate that the results of the model are supported by data from other physical measurements. This is the first quantitative calculation of ionic concentrations in an NPPIL.

## 1 Introduction

Protic ionic liquids (PILs) have been the subject of intensive study in recent decades.^1−4^ PILs are created by the mixture of a Bro̷nsted–Lowry acid and base in the absence of an additional solvent, with ions forming via the equation
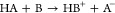
1

The exact definition of a PIL is a complicated question, and a number of schemes for characterizing ionicity and/or defining “ionic” behavior have been explored.^5−8^ Regardless of the scheme used, the difference in p*K*_a_ between the reagent acid and the conjugate acid must be large enough to drive proton transfer to a significant degree if ions are to form.

Researchers have also investigated ionic liquids based on oligomeric anions,^9−11^ described by the equation
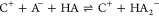
2where C^+^ is a cation incapable of acting as a proton acceptor (e.g., a tetraalkylphosphonium) and A^–^ is the conjugate base of the carboxylic acid HA. The dimer formed by a carboxylic acid and its conjugate base is a known and relatively stable species, and its salts have been studied for many decades.^12^ More recent work^9−11^ has examined the same species in the liquid state. Under these conditions, eq 2 should perhaps be viewed as an equilibrium, in which multiple states can exist,^11^ though Bica and Rogers^10^ have isolated the labile proton signal for a salicylic acid/salicylate dimeric anion, suggesting that it is a major, if not exclusive, anionic component of the liquid.

Other work considers different ionic liquid formulations in which the dimeric anion may be present.^11,13,14^ Consider the reactions
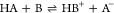
3
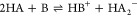
4

This equilibrium is distinct from that in eq 2, in that the cation is formed via proton transfer. In the limiting case where the mole fractions of HA and B are equivalent and the p*K*_a_ for HA is much lower than that for HB^+^, only eq 3 applies (i.e., it is a PIL governed by eq 1). However, if neither of these criteria is met, an equilibrium will establish itself with some population of each of the species in eqs 3 and 4 present. In particular, at high concentrations of acid, the products of eq 4 will be favored.

A variety of approaches have been used to study this case. Ribeiro et al. have studied the azeotropes of acid–base mixtures and have observed them to converge to ratios between 1:1 and 2:1 acid/amine, confirming the particular stability of the anionic dimer.^15^ Berton et al. have studied the spontaneous emergence of a biphasic system, including one acid-rich phase, from the equimolar mixture of acetic acid and triethylamine.^16^ Middendorf and Schönhoff^14^ perform electrophoretic NMR and other physicochemical experiments to characterize charge transport and demonstrate the importance of the formation of anionic dimers in charge transport, where a stoichiometric excess of acid is present. Watanabe et al.^17^ have made spectroscopic and physicochemical studies of carboxylic acid/amide systems and confirmed that at least in some formulations, these systems behave as ionic liquids, albeit poor ones, based on a Walden plot. They further note that the mixtures in which the ratio of acid to amine is greater than unity are better ionic liquids than those in which it is equal to or less than one. This is consistent with the idea that dimeric anions should emerge at high acid concentrations. Watanabe et al.^17,18^ have coined the term “pseudoprotic ionic liquid” for species of this type where the acid/amine ratio is 1:1 and the broader term “nonstoichiometric pseudoprotic ionic liquid” (NPPIL) for other ratios.

These studies represent an offshoot of ionic liquid research; however, earlier strands of investigation consider these systems as well. Kohler et al.^19−21^ study viscosity, conductivity, vapor–liquid equilibrium, and other physical properties of amine/acid mixtures and observe that their physicochemical behavior for such mixtures varies strongly with the acid/amine ratio. The authors attribute the observed changes to the formation of a strong hydrogen-bonding network in the mixture and observe that they generally occur at roughly whole number acid/amine ratios, typically 2:1 or 3:1. Though the authors do not specifically raise the question of the oligomeric anion species, their results are consistent with its emergence in acid-rich domains.

Recent work in our laboratory^22−24^ and others^25−27^ has demonstrated that NPPILs can be used for metal extractions and for other aqueous liquid–liquid extraction processes.^28^ NPPILs also display a novel ability to stabilize the pH of an aqueous phase via extraction of excess acids or bases (i.e., they act as “floating buffers”).^29^ These properties create intriguing possibilities for the technological application of NPPILs.

Our laboratory has also conducted various physicochemical studies,^30,31^ an important aspect of which has been the use of small-angle X-ray scattering (SAXS). We have documented the emergence of nanoscale local liquid structure at compositions corresponding to approximately 1:3 amine/acid ratio for a wide range of amine/acid systems.^30,31^ These observations are complementary to the physical observations of Kohler and co-workers and of Watanabe et al. noted above, in that the novel physicochemical features of pseudoprotic ionic liquids correlate with the emergence of this local structure.

The fact that the observed physicochemical anomalies—including increases in viscosity, conductivity, and nanoscale local liquid structure observable via SAXS—do not correlate exactly with a 1:2 amine/acid ratio suggests that at least for some systems, eqs 3 and 4 should, under most conditions, be viewed as an equilibrium system rather than a reaction that approaches completion. It might also be worth considering whether higher-order oligomeric anions are possible, as has been suggested previously,^13^ which would further complicate the equilibrium.

**Figure 1:**
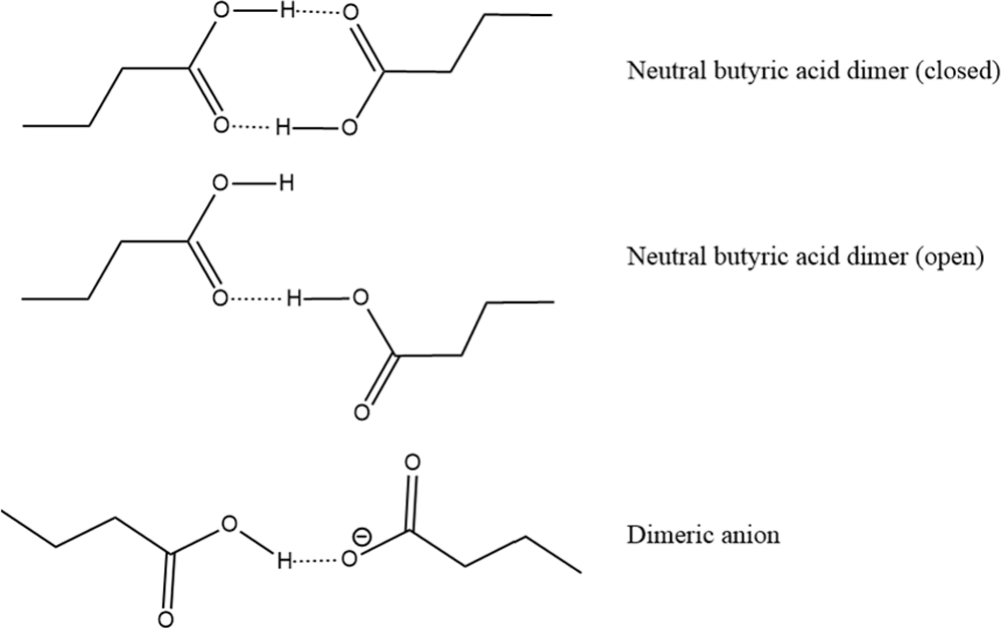
Dimeric species considered in this work. The dimeric structures shown are examples of forms that might exist in the reported mixtures. While the presence of dimers is known, the details of their structure are unconfirmed.

Our goal is to explore the nature of the equilibrium described by eqs 3 and 4 for two sets of mixtures, trihexylamine/butyric acid (T6A/HBA) and water/butyric acid (H_2_O/HBA). The trihexylamine/butyric acid mixture is similar to those considered in prior studies, allowing a comparison to a larger body of research; structures for species that might exist in T6A/HBA are given in Figure 1. The decision to study water/butyric acid mixtures is motivated by a desire for a comparative study with an alternative base. Water is by no means well-understood as a liquid but is a well-studied and structurally simple molecule, making it an appropriate point of comparison with prior work that primarily considers amines and amides. While it may seem odd to incorporate water as a component of an ionic liquid, there is no conceptual reason that a hydronium cation or positively charged water cluster could not be a component of an NPPIL.

We make use of SAXS, infrared spectroscopy, and NMR spectroscopy to characterize the proton transfer equilibrium. For the T6A/HBA mixture, we extend this analysis using a numerical model fitted to ^1^H and ^13^C NMR spectra that make it possible to quantify ionic populations (no such fitting is possible for H_2_O/HBA, see Section 3.4). The results of the NMR-based model are demonstrated to be consistent with those of the other experimental techniques.

## 2 Methods

### 2.1 Sample Preparation

Butyric acid (Aldrich, ≥99%), sodium butyrate (Sigma-Aldrich, 98%), and trihexylamine (Aldrich, 96%) were used as received. Water was HPLC+ grade (Sigma-Aldrich). Samples were prepared in batches of ∼10 mL. Mixtures were prepared by weight, with samples transferred by a syringe to 15 mL centrifuge tubes and subsequently mixed for 5 min at 2000 rpm on a Vortex Mixer (ThermoScientific). Sample compositions ranged from pure base to pure acid in 0.1 increments in the acid mole fraction.

### 2.2 SAXS Experiments

Samples for the SAXS study were transferred to 1.0 mm OD thin-walled borosilicate glass capillary tubes (Charles-Supper) and sealed with hot glue (Superbonder, All Purpose Stik). SAXS measurements were performed on a SAXSLabs (Amherst, MA) custom instrument, equipped with a Bruker AXS copper rotating anode and a PILATUS 300 K area detector. The detector’s nominal position was set at 0.2 m to investigate a range from roughly *q* = 0.05–2.3 Å^–1^; the detector’s actual position was calibrated with silver behenate. The data reduction from 2D to 1D was done with Saxsgui software (saxsgui.com). No separate background subtraction was performed on the data as the background signal associated with the capillaries was roughly 2 orders of magnitude lower than the signal from the sample, and the background signal was flat in the region of interest.

### 2.3 Infrared Spectroscopy

Infrared spectra were recorded in attenuated total reflectance mode at ambient temperature by using a Nicolet iS10 FTIR spectrometer equipped with a Smart iTX accessory.

### 2.4 NMR Spectroscopy

^1^H and ^13^C NMR spectra for mixtures were recorded on a 400 MHz nuclear magnetic resonance spectrometer (Bruker). To avoid any contamination that could disrupt the hydrogen-bonding network of interest, samples were not diluted by solvent, and calibration was obtained using an external reference phase isolated in a flame-sealed capillary NMR tube (Wilmad, 2.5 mm O.D.). This separation also precluded H/D exchange that could influence chemical shifts of the components of the NPPIL. Some proton samples were calibrated using a 36% by mass solution of 3-(trimethylsilyl)propionic-2,2,3,3-d4 acid sodium salt (Sigma-Aldrich, 98% atom D) in deuterium oxide (Aldrich, 99.9% atom D). These calibration standards were adequate but were observed to have a limited shelf life with a decomposition product that led to water that was observed in spectra. This did not interfere with observations and was tolerated; however, subsequent proton spectra made use of a 4% by mass solution of 3-(trimethylsilyl)-1-propanesulfonic acid sodium salt (Sigma-Aldrich, 97%) in deuterium oxide (Aldrich, 99.9% atom D); spectral comparison confirmed no measurable difference in the chemical shift of the silyl group used for calibration. The latter solution was used for all of the ^13^C NMR experiments.

NMR spectra for a number of compounds were recorded to serve as reference spectra supporting the numerical modeling in Section 3.4. These included 10 mM butyric acid in acetone-d6 (Sigma-Aldrich, 99.9%D, 0.03% v/v TMS); sat. sodium butyrate in methanol (Reidel-deHaen, 99.9%; solvent undeuterated); and a mixture of 0.9 mole fraction in T6*A*/0.1 mole fraction in trichloroacetic acid (Sigma-Aldrich, ≥99.0%). Calibration for the last two mixtures used the external standard described above.

The numerical modeling for the NMR data discussed in Sections 3.4 and S5 was performed in Mathematica 13.3 (Wolfram Research), with the coefficients determined using the FindRoot function.

## 3 Results and Discussion

The two mixtures, trihexylamine/butyric acid and water/butyric acid, were studied with compositions ranging from χ = 0 to χ = 1 at increments of 0.1, where χ is the mole fraction of the acid.

### 3.1 Small-Angle X-ray Scattering

**Figure 2:**
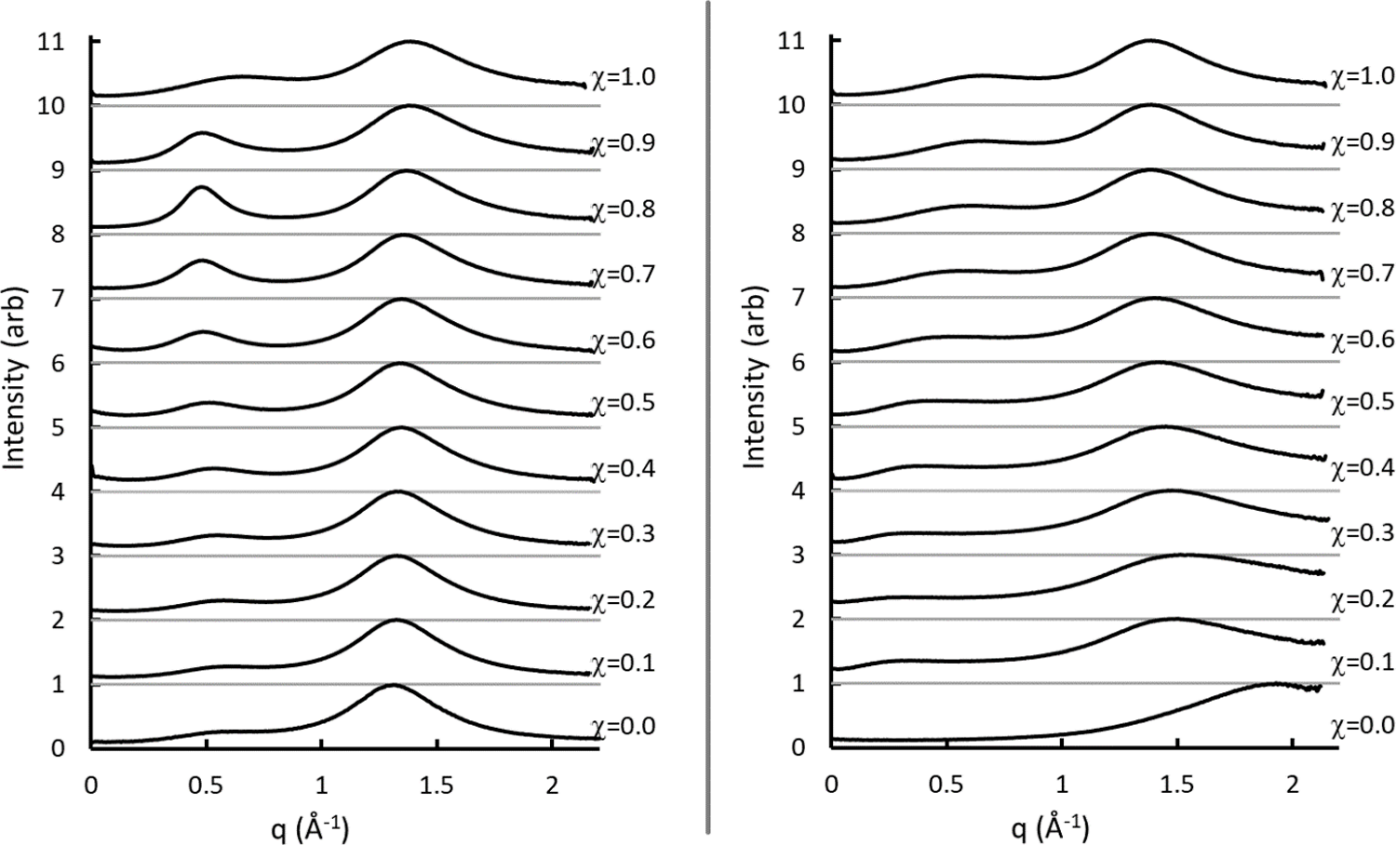
Small-angle X-ray scattering results as a function of composition. Left: T6A/HBA. Right: H_2_O/HBA.

Small-angle X-ray scattering (SAXS) profiles are given in Figure 2 for the T6A/HBA and H_2_O/HBA mixtures as a function of composition. Looking first at T6A/HBA and beginning with the pure acid (χ = 1), the results reported here are similar to those reported elsewhere for linear carboxylic acids.^31−33^ An inter/intramolecular peak associated with the alkyl chains is visible near *q* = 1.4 Å^–1^, and a broad peak known to correspond to hydrogen-bonded acid dimers is visible near *q* = 0.7 Å^–1^. This latter peak is associated with the formation of hydrogen-bonded acid dimers, and its position depends strongly on the length of the alkyl chain of the acid. The configuration underlying the peak most likely represents acid molecules arranged tail-to-tail, forming hydrophilic and hydrophobic domains. Simple geometric arguments imply that a single butyric acid molecule should have a length of ∼4 Å, and the peak reported here with a correlation length of ∼9 Å is consistent with its interpretation as a dimer. Prior studies report similar relationships between the acid length and local structure peak.^33^

Examining the χ < 1 cases for T6A/HBA, it is easy to see that the dimerization peak narrows and it moves to lower *q*, corresponding to an increase in nanoscale structure length scale as χ decreases to ∼0.7. This is consistent with observations of a wide range of acid/amine systems discussed in prior work^31^ and is associated with the formation of a charged dimer or oligomer along the lines indicated by eq 4. A similar though less pronounced peak is observable in H_2_O/HBA; however, there are qualitative differences between the two mixtures that will be discussed below.

**Figure 3:**
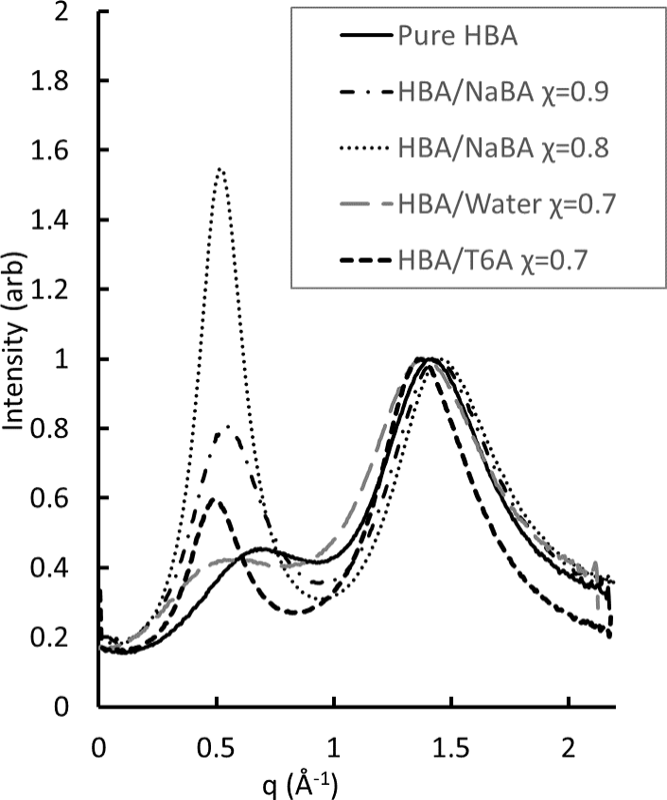
Comparison of local structures for pure butyric acid to H_2_O/HBA and T6A/HBA mixtures. Mixtures of butyric acid and sodium butyrate are included for reference; see the text for discussion.

Figure 3 offers insight into the nature of the observed structure for χ < 1. The figure includes pure butyric acid and the χ = 0.7 SAXS results for both T6A/HBA and H_2_O/HBA. The shift to lower *q* values for the mixtures is evident. Also included in Figure 3 are signals for mixtures of sodium butyrate (NaBA) and butyric acid in different compositions. The peak centers for the H_2_O/HBA, T6A/HBA, and NaBA/HBA mixtures are very similar, and the peak height increases as the mole fraction of NaBA increases in the NaBA/HBA mixtures (i.e., goes from χ = 0.9 to 0.8). These observations confirm the presence of the dimeric anion in all three cases, as there is no other structure that could plausibly exist in all three mixtures that would not also be present in the pure acid.

The increase in the intensity of the local structure peak with an increasing NaBA concentration is striking. It is unclear whether this arises because of an increase in the population of dimeric anionic species relative to the T6A- and H_2_O-containing species or whether the presence of the sodium ion is in some way altering the scattering cross-section. As this is of secondary interest to the present study, we do not investigate further.

The nanoscale structure of the T6A/HBA system becomes less pronounced as χ decreases below 0.7, and the correlation length (structure size) starts to decrease and approaches the structure of the pure amine for χ < 0.5. In the H_2_O/HBA system, there is no enhancement of the nanoscale structure with decreasing χ; however, there is a gradual shift of the peak position to lower *q* (corresponding to a longer length scale for the structure) that extends to very low *q* values at χ = ∼0.3. Numerical fitting is suspect without a clearer understanding of the structure; however, a naive fitting to the χ = 0.2 and 0.3 data sets detailed in the Supporting Information gives the center of the local structure peak at *d* = 20.8 and 16.0 Å, respectively (see Section S1). Intriguingly, these length scales exceed the largest possible physical dimensions of the acid dimers. We have not observed such a signal in studies of acid/amine mixtures; we will discuss it in Section 3.2, as further insight can be obtained from infrared spectroscopy.

### 3.2 Infrared Spectroscopy

Infrared spectra for T6A/HBA and H_2_O/HBA are given in Figure S.3 in the Supporting Information. The infrared spectra of acids and their conjugate bases have been studied extensively,^9,34,35^ and specific features offer insight into the character of the species present in solution. Figure S.3 shows that spectral features vary smoothly from χ = 0.0 to χ = 1.0. Therefore, it is productive to focus on a limited set of compositions for interpretation; we choose χ = 0, 0.3, 0.7, and 1.0 for this purpose.

**Figure 4:**
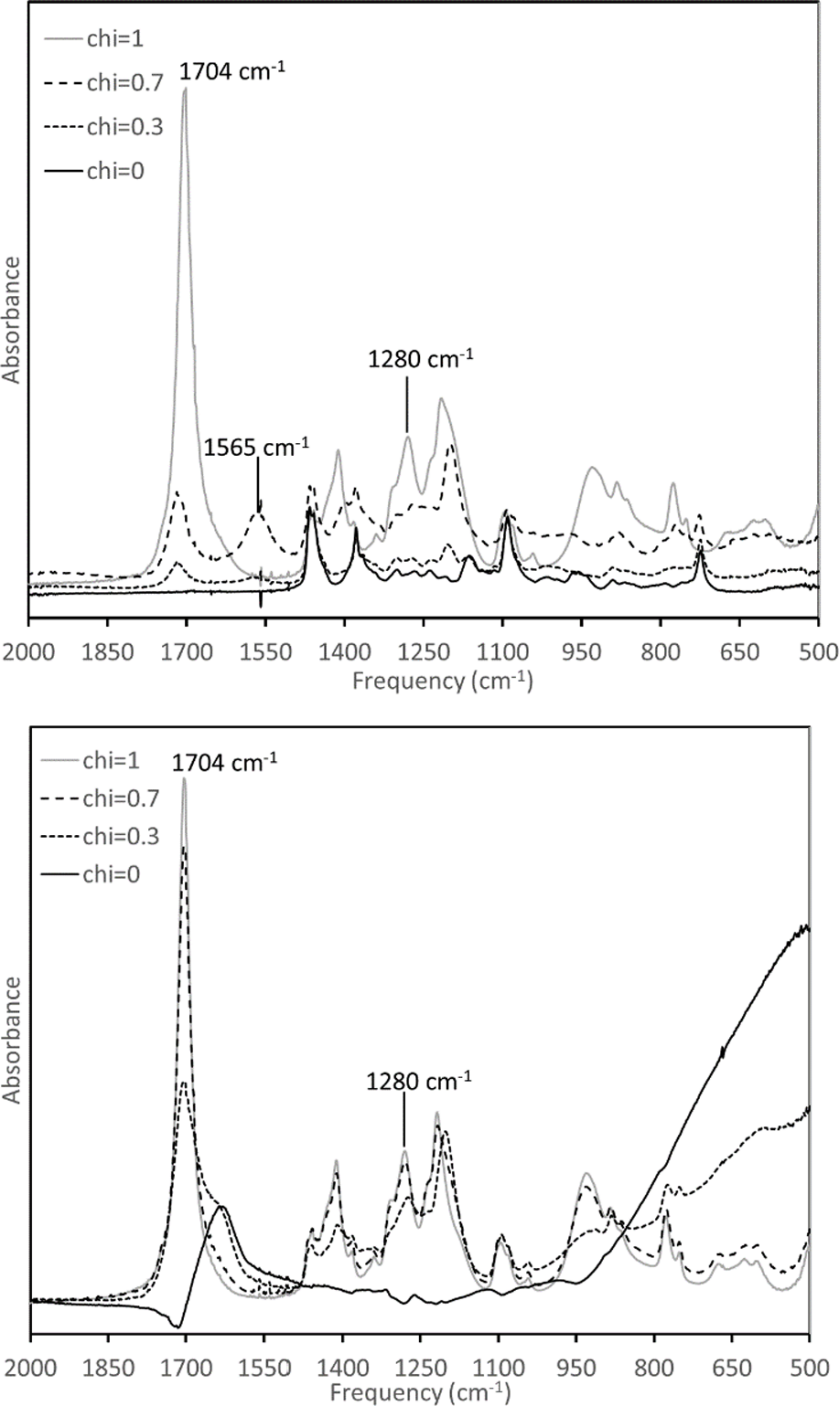
Infrared spectrum of T6A/HBA (top) and H_2_O/HBA (bottom).

Figure 4 shows a close comparison for χ = 0.0, 0.3, 0.7, and 1.0 in the range of 400–2000 cm^–1^. Our analysis follows the example of Berton et al.,^9^ who considered infrared spectroscopy of a mixture of acetic acid and *n*-butylamine. These authors note that the acetate anion displays an asymmetric stretch at 1534 cm^–1^ that splits into peaks at 1712 and 1280 cm^–1^ when the anion is protonated, similar to earlier observations in dilute aqueous solution.^35^ Analogs to the latter peaks are present in the butyric acid spectrum at 1704 and 1280 cm^–1^, and these peaks are visible to lesser degrees for the χ = 0.3 and 0.7 T6A/HBA mixtures, indicating the presence of the protonated acid. However, these compositions also show a peak at 1565 cm^–1^, likely analogous to the asymmetric stretch of the acetate anion and implying significant deprotonation.

No peak is visible near 1565 cm^–1^ in the H_2_O/HBA mixtures, and the peaks at both 1712 and 1280 cm^–1^ are relatively intense. This suggests that the population of molecular acid may be higher than that observed in analogous compositions of T6A/HBA, with the population of anionic dimers being relatively low. This would be consistent with the reduced intensity of the SAXS peak attributed to anionic dimers, as discussed in Section 3.1.

**Figure 5:**
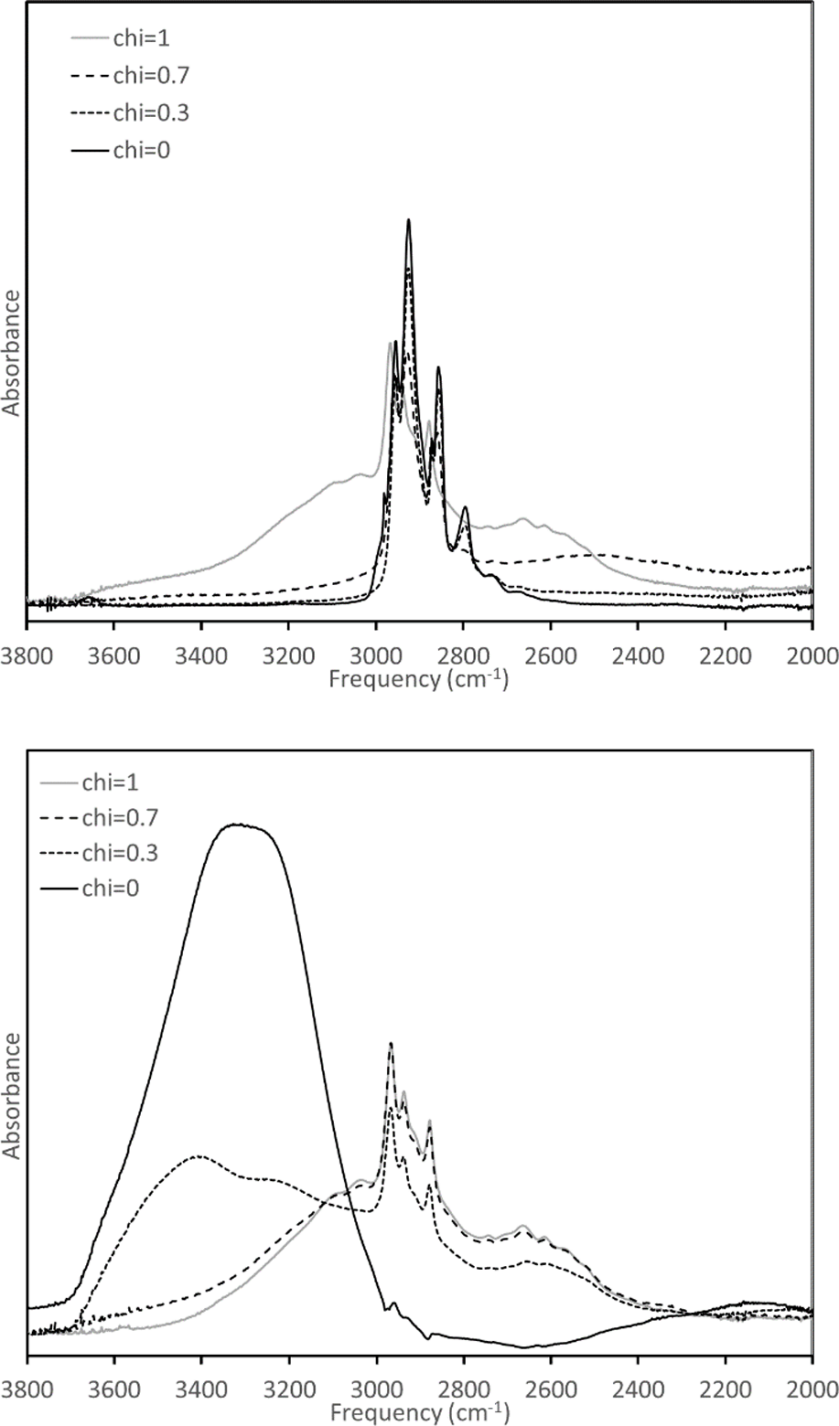
T6A/HBA (top) and H_2_O/HBA (bottom). See the text for discussion.

Figure 5 examines the high-frequency region of the spectrum. Early work by Odinokov et al.^36,37^ and more recent studies^38,39^ indicate that the region above 3000 cm^–1^ is associated with “free” O–H motions indicative of weak or absent hydrogen bonding to neighboring species. As the strength of the hydrogen bond increases, peaks generally red shift owing to a lower effective force constant for the bond. Work by Van Hoozen and Petersen^38^ indicates that for acids hydrogen-bonded to bases, this red shift is associated with a narrowing of the spectrum. In broad strokes, this is consistent with the observations in the current study, as the T6A/HBA spectrum shows a spectral narrowing and a slight red shift for χ = 0.7 and 0.3 relative to the pure acid. Such effects are far more limited for the H_2_O/HBA mixture, consistent with the lower basicity of water relative to T6A.

This analysis suggests that the broad, featureless band near 2500 cm^–1^ in T6A/HBA for χ = 0.7 should be indicative of a hydrogen-bonded complex. This complex is not observed in the H_2_O/HBA mixtures, nor is it present for pure T6A or HBA; its signal might be very weakly visible in T6A/HBA for χ = 0.3. This could correspond to hydrogen bonding of the anionic dimer observed by SAXS at the same composition. However, this assignment is speculative and, in any case, is not central to the thrust of this report.

Looking at the features near 3400 cm^–1^ for the H_2_O/HBA system, the maximum for the χ = 0.3 case is slightly blue-shifted relative to pure water. Brubach et al.^40^ reported a similar blue shift in the infrared spectra for water confined in reverse micelle systems, and some form of confinement may be at work here. This is interesting in light of the SAXS results, suggesting the existence of a nanoscale local structure for mixtures with this composition at length scales exceeding the maximum molecular dimensions of acid dimers.

We explore this possibility by making a numerical fit of the infrared spectrum. Brubach et al.^40^ fit their data using a sum of three Gaussian curves, which they assign to specific classes of water (“network,” “intermediate,” and “multimer”). Our spectrum is complicated by the overlap of the aqueous signal with the blue wing of butyric acid. To compensate for this, we fit the pure butyric acid spectrum in the corresponding region to a Gaussian curve and incorporate this information into a subsequent fit of the χ = 0.3 spectrum. In this latter fit, the data were described by the sum of three Gaussians plus the fitted butyric acid peak; the amplitude of the last peak was varied as part of the fit, but the center and width were fixed. The results are given in Figure S.6; details of the fit are reported in the Supporting Information (see Section S3).

Table 1 compares the centers of the Gaussians determined in this work to those of Brubach et al. The network water is a relatively poor match, with a difference of 74 cm^–1^ in the peak centers. However, this may be an artifact of the overlap between this peak and the blue wing of butyric acid. The other features are both within 20 cm^–1^ of their analog in the Brubach study, suggesting a similar physical origin.

**Table 1:** Peak Centers for Numerically Fitted Data in Brubach et al.^40^ and This Work^a^

feature	Brubach et al.	this work
network	3310 cm^–1^	3236 cm^–1^
intermediate	3431 cm^–1^	3455 cm^–1^
multimer	3580 cm^–1^	3570 cm^–1^

a See the text for discussion.

It is worth noting that butyric acid is the heaviest linear carboxylic acid that is miscible with water; pentanoic acid, while very soluble in water, phase separates at high concentrations. It is possible that the butyric acid/water liquid structures at smaller *q* may be viewed as nanoscale segregation of the liquid environment into more hydrophobic regions with tail-to-tail arrangement of aliphatic chains and water-rich regions incorporating the acid head groups.

This is consistent with the SAXS results discussed in Section 3.1. The length scale for the local liquid structure is roughly that of a dimer for χ > 0.6; however, as water content increases at low χ, the length scale increases to ∼21 Å, most likely due to increased separation between hydrophobic domains. Intriguingly, the increase in length scale between χ = 0.7 and 0.3 is approximately equal to the thickness of a hydration layer, suggesting the formation of a hydrogen-bonding network linking water and acid headgroups rather than the carboxyl-to-carboxyl bridging implied by the schematic in Figure 1. There is no evidence of a form factor in the SAXS data at low *q*, indicating a relatively homogeneous distribution of small domains separated by a hydration layer rather than the discrete aggregates that fatty acids of higher molar mass are known to form.^41^ While intriguing, these questions are secondary to the proton transfer phenomena that are central to the present work, and we reserve their investigation for future work.

### 3.3 Nuclear Magnetic Resonance Spectroscopy

**Figure 6:**
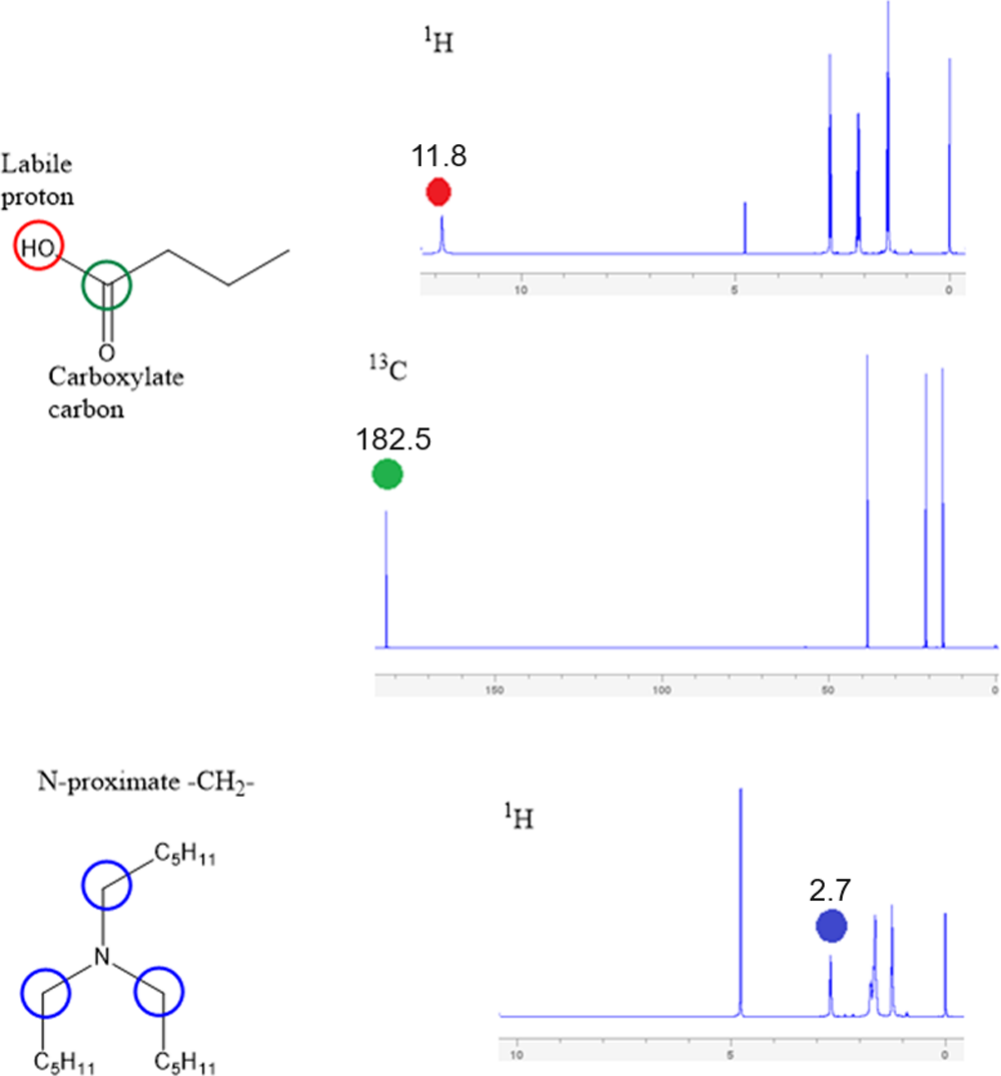
^1^H and ^13^C NMR assignments are for peaks that are sensitive to the protonation and dimerization states of the mixture. The top two spectra are taken from pure samples of butyric acid, and the bottom spectrum is from pure trihexylamine.

We characterized the NPPIL mixtures using both ^1^H and ^13^C NMR spectroscopy. NMR spectra for pure HBA and T6A are given in Figure 6. NMR spectra are known for butyric acid^42^ and trihexylamine,^43^ and we do not review these assignments in detail here. ^1^H and ^13^C NMR spectra for the H_2_O/HBA and T6A/HBA mixtures in all compositions are given in Section S4, and for the most part, these spectra are readily interpreted as sums of the spectra of the component molecules. Only those chemical shifts affected by the dimerization and protonation states of the acid require further analysis. These chemical shifts are identified in Figure 6.

**Figure 7:**
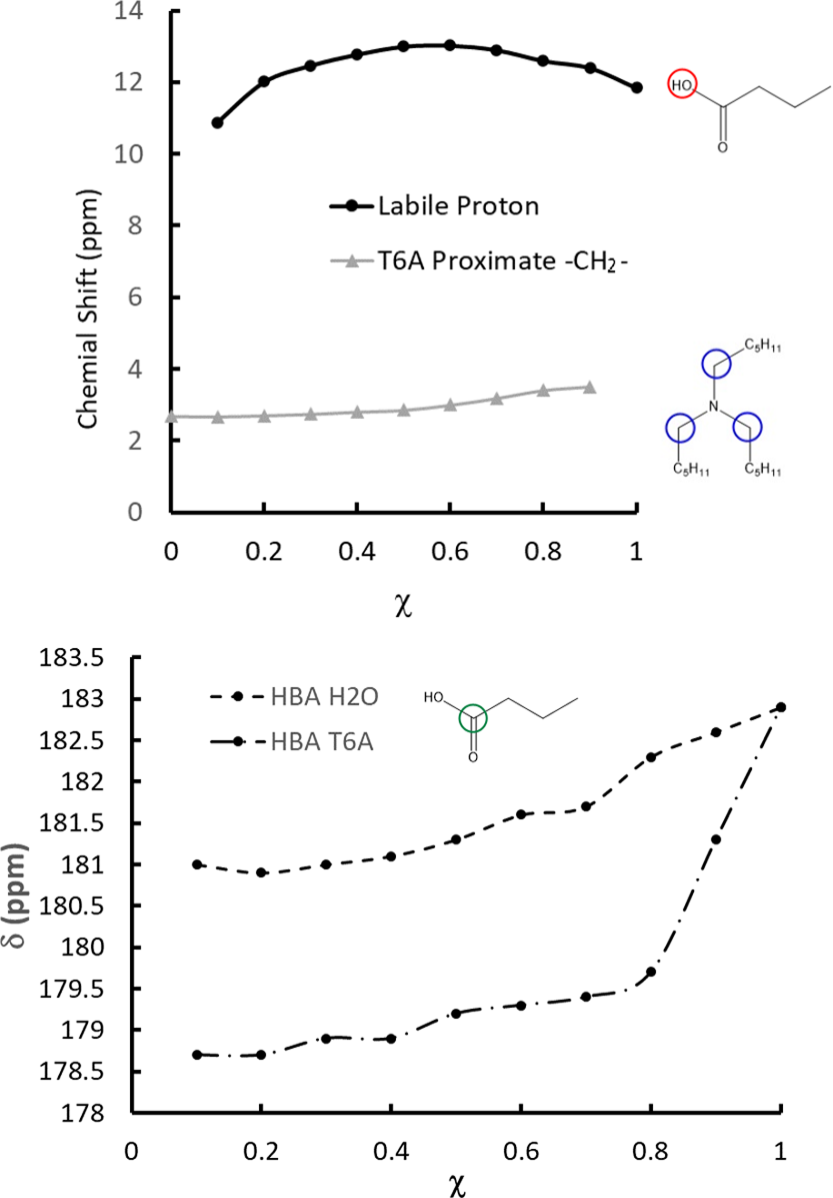
Top: Chemical shift of the labile proton and the T6A N-proximate −CH_2_– groups as a function of χ for the T6A/HBA system. Bottom: Chemical shifts of carboxyl carbon were observed in H_2_O/HBA and T6A/HBA systems.

The chemical shift for the labile proton in the T6A/HBA mixture as a function of χ is reported in Figure 7. As T6A is added to the system, the chemical shift increases, reaching a maximum at χ = 0.6 before decreasing at lower χ values. This increase arises from the fact that deprotonation of the acid leads to the formation of a protonated amine, and at high acid concentrations, it may also lead to the creation of a dimeric anion, as given in the equation below (analogous to eq 4)
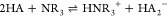
5

Assuming no monomeric anion is formed, the chemical shift of the labile proton is thus a weighted average over all of the molecules present in eq 5. Making use of this insight requires knowledge of the chemical shift of the labile proton for the protonated amine (HNR_3_^+^) and anionic dimer (HA_2_^–^).

We determined the chemical shift of the labile proton on the amine in a mixture of 0.9 mole fraction in T6A and 0.1 mole fraction in trichloroacetic acid, observing a value of δ = 8.8 ppm. The large stoichiometric excess of the amine and the relative strength of trichloroacetic acid (p*K*_a_ = 0.70)^44^ dictate that acid ionization should be nearly complete. Given that trichloroacetic acid is the only source of labile protons in this mixture, the observed chemical shift must correspond closely to that of the labile proton of the protonated amine.

Bica and Rogers^10^ have isolated the labile proton signature for the salicylic acid/salicylate anionic dimer and have observed a chemical shift of 19.2 ppm. Such isolation is possible only in liquids with exceptionally high viscosities (and correspondingly slow hydrogen ion exchange rates) and, therefore, is not observed in the present system. However, if the butyric acid/butyrate anion has a comparable chemical shift, the average chemical shift associated with the products in eq 5 will drive the chemical shift upfield.

The observation that the labile proton chemical shift increases until χ = 0.6 may seem inconsistent with the observation that the SAXS intensity associated with the dimer is greatest at χ ∼ 0.8; however, there is no contradiction. The SAXS results relate to the absolute population of the dimer, as this determines scattering intensity, while the chemical shift in Figure 7 correlates with the relative populations of the protonated amine and dimeric anion. The result is therefore possible if the absolute population of dimers decreases even as the relative proportion of deprotonated acid molecules increases below χ = 0.8. We will return to this point in Section 3.4.

The decrease in chemical shift for χ < 0.6 does not necessarily mean that deprotonation has decreased; it may simply mean that at lower concentrations of acid, a monomeric anion is favored over the presence of a dimeric one. Indeed, for χ = 0.1, the chemical shift drops below that of the pure acid, which is possible only in the presence of a population of protonated amines that are not balanced by the presence of a corresponding dimeric anion.

Further insight can be gleaned by inspecting the chemical shift of the amine −CH_2_– hydrogens on the carbon proximate to the amine nitrogen. In pure T6A, this corresponds to a chemical shift of 2.7 ppm, which is the same value as is observed for χ = 0.1, indicating that there is negligible protonation of the amine. This is consistent with the labile proton chemical shift at χ = 0.1, which, at 10.9 ppm, is less than that of the pure butyric acid case at χ = 1.0. This is only possible if the extent of deprotonation of the acid is very small, and the neutral dimers that are prevalent in the pure acid are largely absent, with neutral butyric acid monomers being the dominant form. In dilute solution in acetone, the observed butyric acid chemical shift is 10.4 ppm (see the Supporting Information), which is less than the 11.8 ppm observed for pure butyric acid. This likely reflects the difference in chemical shift between monomeric and dimeric acid forms.

The information obtained from ^1^H NMR spectroscopy can be supplemented by the ^13^C NMR results. Cistola et al.^45^ have demonstrated that the protonation state of small linear carboxylic acids (including butyric acid) can be inferred from the chemical shift of the carboxyl carbon in ^13^C NMR. The authors observe that the chemical shift increases by roughly 5.0 ppm over the course of deprotonation. In separate experiments, the authors also report the chemical shift as a function of the concentration of butyric acid in water, ranging between 0.1 and 1.0 mole fraction in acid while maintaining a constant pH of 2.5; with deprotonation suppressed, this shift must reflect dimerization. Thus, the chemical shift increases both with deprotonation and with increasing concentration.

Carbon-13 NMR spectra for the T6A/HBA mixtures are given in Section S4. The chemical shift of the carboxyl carbon is reported in the bottom frame of Figure 7; it decreases rapidly from χ = 1.0–0.8, at which point the slope flattens significantly. The modeling discussed in Section 3.4 suggests the decrease as χ drops to 0.8 is associated with an increase in the number of anionic dimers and a decrease in the population of neutral dimers. The flatness for χ < 0.8 is associated with a relatively constant distribution of acid species below that value.

Attempting a similar analysis for the H_2_O/HBA system is less informative. As observed in prior studies of carboxylic acid/water mixtures at high acid concentrations,^46,47^ adding water to the system increases the population of labile protons such that the chemical shift of the labile proton is dominated by water. A further complication is that, as suggested by the IR and SAXS data, some of the water may be confined, which would alter its chemical shift.^48^ The above techniques do not offer insight into the relative populations of “confined” vs “free” water, eliminating any obvious route to the analysis of the contributions of the two states. In light of this, the ^1^H NMR spectra for the H_2_O/HBA mixtures are reported in Section S4; however, we do not attempt a detailed analysis.

The ^13^C spectra are presented in Section S4, with the chemical shift of the carboxyl carbon reported in Figure 7. The results are in general agreement with the results of Cistola et al.,^45^ though the experiments are not precisely analogous, as those authors maintained either constant pH or constant acid concentration, while in the present case, those properties vary concurrently. The observed trend is similar to that for T6A/HBA, although the less pronounced decrease in chemical shift may be associated with a lower basicity for water.

### 3.4 Numerical Modeling of NMR Data

With these ideas in mind, we modeled the NMR data and inferred the populations of different species in solution. We identify four possible states for the acid and assign a mole fraction to each: n_HA_ denotes the population of neutral monomers, n_A_ denotes the population of anionic monomers, n_H2A2_ denotes the population of neutral dimers, and n_HA2_ denotes the population of anionic dimers. The mole fractions are constrained by the relationship:
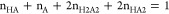
6

The model excludes the possibility of higher-order charged or neutral oligomers; however, this should be a passable approximation if the dimers are the dominant form.

With these definitions, we may write the chemical shifts for the labile proton and the carboxyl carbon atom as
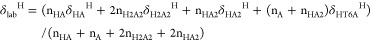
7
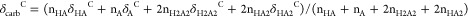
8where δ_i_^X^ is the chemical shift of the i atom of element X.

If the chemical shifts of the different species are known, eqs 6–8 represent a set of three algebraic equations corresponding to four unknowns (the n_X_ values). For the H_2_O/HBA mixtures, this leaves the acid mole fractions underdetermined, and we did not pursue this analysis. However, for the T6A/HBA mixtures, the hydrogens in the nitrogen-proximate −CH_2_– groups are sensitive to the protonation state of the amine (see Figure 7). This creates an additional relationship
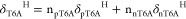
9where pT6A and nT6A correspond to the protonated and neutral forms of the amine. Since all protons in the system are donated by the acid, the chemical shift can be calculated from the populations of deprotonated acid:
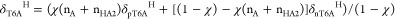
10

This presents a fourth algebraic equation and makes it possible to determine the acid mole fractions n_HA_, n_A_, n_H2A2_, and n_HA2_ for the T6A/HBA mixture.

The reference chemical shifts for the δ_i_^X^ terms in eqs 6–10 can be determined experimentally. This can be done either by characterizing T6A or C4A outside of an NPPIL, as was done to determine the chemical shift for protonated T6A (δ_HT6A_^H^) based on a mixture of 0.9 mole fraction T6A/0.1 mole fraction trichloroacetic acid, described in Section 3.3. In other cases, it can be inferred as a limiting case for the NPPIL system, for example, by inferring the chemical shifts of the neutral dimer (δ_H2A2_^H^ and δ_H2A2_^C^) from the spectrum of the pure acid (i.e., assuming that the only species present was the neutral dimer). The details of these reference chemical shift determinations are given in Section S5.

Using values determined in this way to fit eqs 6–8 and 10 is expected to lead to imperfect outcomes for several reasons. First, both ^1^H and ^13^C chemical shifts depend on the solvent, and the different solvent environments used to obtain the reference chemical shifts are not fully consistent with each other. This problem is exacerbated by the fact that the character of the solvent in the T6A/HBA mixture changes as χ ranges from 0 to 1.0. Further, the chemical shift of the labile proton and that of the carboxyl carbon depend on the relative p*K*_a_ values of a hydrogen-bonded donor/acceptor pair,^49,50^ creating a further discrepancy between the reference state and NPPIL. Finally, as noted above, Cistola et al.^45^ reported that the ^13^C chemical shift of the carboxyl carbon varies with concentration, a property that changes with χ; this dependence should, in principle, be accounted for in the relative values of n_HA_ and n_H2A2_, though this assumes a linear relationship. However, while the analysis must be viewed with some caution, the results of the fitting process can be evaluated for their physical consistency and compared to the results of SAXS and infrared spectroscopy. Despite the limitations of this approach, we are not aware of any other analysis that allows simultaneous quantification of four different chemical species that differ only in their protonation and hydrogen bonding states.

**Figure 8:**
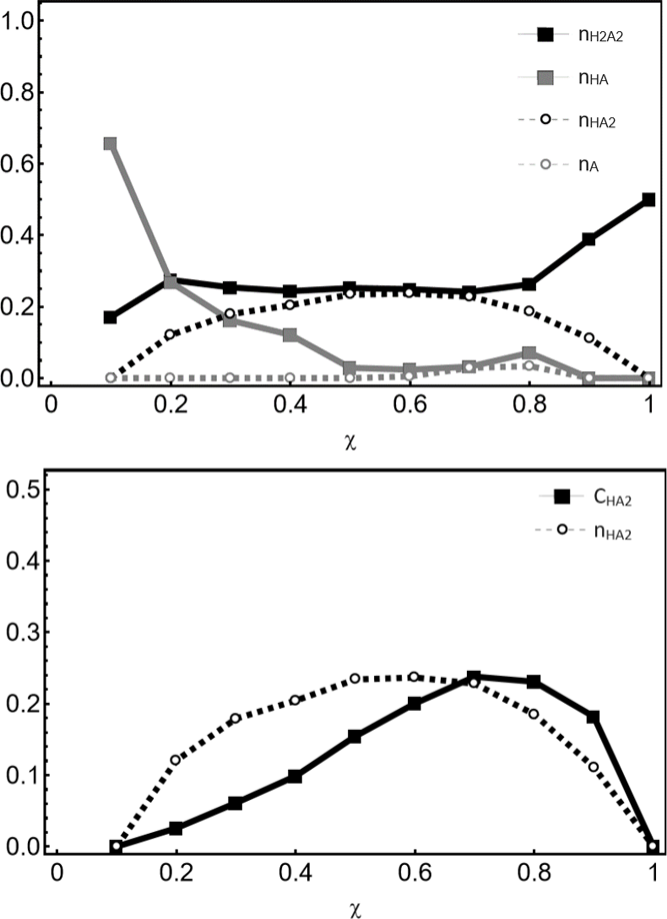
Top: Coefficients for neutral (squares) and charged (circles) species in the T6A HBA mixtures. See the text for discussion. Bottom: Mole fraction of anionic dimers (n_HA2_) and total population of anionic dimers (C_HA2_) vs acid population. See the text for discussion.

Details of the fitting process are given in Section S5. The fitted functions reproduce the observed NMR shifts with a high degree of accuracy for χ > 0.1, though for χ = 0.1, there are inconsistencies between ^1^H and ^13^C that lead to a spurious result for the fit (see Section S5 for discussion). The n_*x*_ values obtained from the model are shown in the top frame of Figure 8 as a function of χ. The results suggest that the dimeric anion remains the dominant form of the ionized acid population over the full range of conditions studied. Below χ = 0.5, the concentration of the neutral monomer increases as the concentration of the ionized dimer decreases.

With the construction of our reference states, pure butyric acid (χ = 1) consists entirely of the neutral dimer. Also, for χ = 0.9, the system is taken to have fully ionized all T6A present, and all ionized acid molecules are assumed to have dimerized. The nonzero values for n_A_ at χ = 0.8 and 0.7 suggest that the latter constraint may have been too severe; however, the extent of the error cannot be inferred within the available data. Nevertheless, the behavior is broadly consistent with physical expectations, with dimeric forms dominating at high χ and the neutral monomer becoming important at low χ. While it might be expected that the presence of a large excess of basic T6A at low χ should drive ionization via Le Chatelier’s Principle, the strength of the monomeric butyrate base operates against ionization, as does the low polarity of a solvent mixture in which T6A is the majority component.

Recall that the n_HA2_ values reported in the top frame of Figure 8 correspond to mole fractions of the dimeric anion relative to the population of acid present in solution, normalized per eq 6. To convert this to a total mole fraction of the species present, including the amine, we write
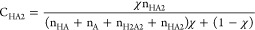
11

This is slightly different from the normalization convention used in eq 6, as it treats dimers as single particles rather than weighting acid species by the number of molecules they contain. However, this convention represents a more appropriate approach when considering the entire population of species present, and the value of C_HA2_ represents the total population of dimeric anions as a fraction of the total number of particles in solution.

The bottom frame of Figure 8 compares C_HA2_ and n_HA2_. C_HA2_ peaks at χ = 0.7, a higher value than that of n_HA2_. This reflects the fact that the absolute population of acid decreases with decreasing χ. A review of the SAXS profiles in Figure 2 shows that the local structure peak reaches the maximum intensity for χ = 0.8, following a trend similar to that of C_HA2_. This is sensible because, as discussed in Section 3.1, the local structure feature in the SAXS profile is associated with the oligomeric anion and its behavior should correlate with the absolute concentration of the anionic dimer. The agreement between the NMR-derived model and the SAXS data represents strong experimental evidence for the validity of the model.

Infrared spectroscopy provides another point of comparison with the experiment. If it is assumed that the dimeric anion displays the asymmetric stretch associated with the deprotonated carboxyl group, then the emergence of the feature at 1565 cm^–1^ for χ = 0.7 shown in Figure 4 is also consistent with the results of the model. This feature is comparable in magnitude to the peak at 1704 cm^–1^, which would be expected to be associated with the protonated forms; this is consistent with the indication in Figure 8 that protonated and deprotonated acid forms coexist at χ = 0.7. In contrast, an inspection of the χ = 0.3 trace in Figure 4 indicates that the 1704 cm^–1^ peak is significantly larger than that at 1565 cm^–1^, suggesting the population of protonated acid should be larger than that of deprotonated species. This is also consistent with Figure 8, if one assumes that both the monomeric and the dimeric neutral forms will contribute to the 1704 cm^–1^ signal. Taken together, the SAXS data and the results of infrared spectroscopy provide independent validation for the NMR-derived model.

We are not aware of other published reports that quantify ion populations in NPPILs of this type. Simulation studies are available; however, they consider acetic acid/methylimidazolium systems that are quite different than those studied here.^51,52^ While it is difficult to make a comparison, there is nothing in these studies to suggest inconsistency with the results presented here.

## 4 Conclusions

In comparing the results of the H_2_O/HBA and T6A/HBA experiments, the evidence indicates that less acid ionization is present for the water mixtures, likely reflecting the lower basicity of water relative to T6A. However, the local structure visible in the SAXS results for χ > 0.5 in the H_2_O/HBA mixtures confirms that ionization occurs to some degree and leads to the same local nanoscale structure that is observed in T6A/HBA, indicative of a dimeric anion. The “confined water” behavior suggested by both SAXS and infrared spectroscopy at low χ suggests the emergence of collective structures in the water mixture, perhaps arising from the smaller size and greater polarity of the water molecules relative to the amine. We do not pursue a detailed analysis here; however, there may be interesting connections to the aggregation phenomena associated with higher molecular weight acids and other amphiphiles.^53−55^

For the amine/acid system, the analysis presented here supports a deeper understanding of the nature of ionization in NPPILs. Based on the numerical analysis of NMR data, there is no composition for which the concentration of ionized species in T6A/HBA approaches 100%, meaning that the mixture falls short of the most straightforward definition of an ionic liquid. This is particularly true at high and low amine contents, where the absolute concentration of ions is quite low. It is also worth noting that, as we have inferred that the ionicity of the H_2_O/HBA mixtures is significantly lower than that of T6A/HBA, it would likely be unproductive to label the H_2_O/HBA system as an NPPIL.

However, this does not mean that ionization can be ignored when considering NPPIL behavior. Many of the reports of novel physicochemical features referenced in Section 1 are most evident near χ = 0.8 and are hypothesized to arise from NPPIL ionization. For example, the correlated maxima in viscosity and conductivity observed by Kohler et al.,^20,21^ Johansson et al.,^13^ and by ourselves in earlier work^30^ emerge near a ratio of χ = 0.8 for a wide range of amine/acid mixtures. Assuming the populations of dimeric species in these systems are similar to those observed here, these correlations can be understood as the emergence of hydrogen-bonding networks incorporating neutral and anionic dimers; high populations of both are documented in Figure 8 for χ = 0.8. The emergence of increased hydrogen bonding and inter-ion Coulomb interactions increases viscosity, while the liquid structure facilitates charge transport through the rearrangement of hydrogen bonds and covalent bonds, as is observed in the Grotthuss mechanism in water.^56^

In addition to quantifying ion populations, the description of equilibrium presented here offers important insights. First, the dimeric anion remains the dominant conjugate base in the proton transfer reactions of NPPILs even to relatively low mole fractions in the acid. Second, the emergence of maximum ionization near χ = 0.8 likely reflects a trade-off between the thermodynamic driving force for acid ionization, which increases in the presence of excess acid, and the stoichiometric limits for proton transfer associated with decreasing amine concentration. The trends in the populations of different species vs composition may be important in interpreting other phenomena, such as the azeotropes studied by Ribeiro et al.^15^

The results of these studies connect in interesting ways to studies of PILs. Zhang et al.,^57^ for example, studied the 1-methylimidazolium/acetic acid PIL system and observed an ionicity of roughly 93% for the neat PIL. However, dissolution in solvents changes the degree of ionization, and it is possible that some of the observed concentration-dependent ionization may reflect dimerization of acid species. Likewise, it might be useful to adapt some of the techniques used to study ionicity in PILs to NPPILs, such as dielectric relaxation spectroscopy^58,59^ or the joint calorimetric and spectroscopic approach of Yaghini et al.^60^ The methodology presented here is flexible and can be adapted to the investigation of other NPPILs, meaning it can both support comparative studies of NPPILs and supplement other experimental approaches. Both of these approaches offer exciting new avenues for the study of NPPILs.

## Supporting Information Available

Details of the fitting process used to analyze the low χ H_2_O/HBA SAXS data in Section 3.1 (discussed in Section 3.2); fitting process for the infrared spectroscopy described in Section 3.2; complete set of infrared spectra and NMR spectra for mixtures explored in this study; and details of the fitting process used to determine ion populations as described in Section 3.4, including the NMR spectra for reference states used (PDF)

## References

[ref1] Fadeeva Yu. A.; Gruzdev M. S.; Kudryakova N. O.; Shmukler L. E.; Safonova L. P. Physico-Chemical Characterization of Alkyl-Imidazolium Protic Ionic Liquids. J. Mol. Liq. 2020, 297, 111305 10.1016/j.molliq.2019.111305.

[ref2] Greaves T. L.; Drummond C. J. Protic Ionic Liquids: Evolving Structure–Property Relationships and Expanding Applications. Chem. Rev. 2015, 115 (20), 11379–11448. 10.1021/acs.chemrev.5b00158. 26426209

[ref3] Stettner T.; Balducci A. Protic Ionic Liquids in Energy Storage Devices: Past, Present and Future Perspective. Energy Storage Mater. 2021, 40, 402–414. 10.1016/j.ensm.2021.04.036.

[ref4] Bailey J.; Byrne E. L.; Goodrich P.; Kavanagh P.; Swadźba-Kwaśny M. Protic Ionic Liquids for Sustainable Uses. Green Chem. 2024, 26 (3), 1092–1131. 10.1039/D3GC03297C.

[ref5] Yoshizawa M.; Xu W.; Angell C. A. Ionic Liquids by Proton Transfer: Vapor Pressure, Conductivity, and the Relevance of DeltapKa from Aqueous Solutions. J. Am. Chem. Soc. 2003, 125 (50), 15411–15419. 10.1021/ja035783d. 14664586

[ref6] Hasani M.; Amin S. A.; Yarger J. L.; Davidowski S. K.; Austen Angell C. Proton Transfer and Ionicity: An ^15^N NMR Study of Pyridine Base Protonation. J. Phys. Chem. B 2019, 123 (8), 1815–1821. 10.1021/acs.jpcb.8b10632. 30779573

[ref7] Stoimenovski J.; Izgorodina E. I.; MacFarlane D. R. Ionicity and Proton Transfer in Protic Ionic Liquids. Phys. Chem. Chem. Phys. 2010, 12 (35), 10341–10347. 10.1039/c0cp00239a. 20601995

[ref8] Mann S. K.; Brown S. P.; MacFarlane D. R. Structure Effects on the Ionicity of Protic Ionic Liquids. ChemPhysChem 2020, 21 (13), 1444–1454. 10.1002/cphc.202000242. 32445198

[ref9] Berton P.; Kelley S. P.; Wang H.; Myerson A. S.; Rogers R. D. Separate Mechanisms of Ion Oligomerization Tune the Physicochemical Properties of N-Butylammonium Acetate: Cation-Base Clusters vs Anion-Acid Dimers. Phys. Chem. Chem. Phys. 2017, 19 (37), 25544–25554. 10.1039/C7CP04078D. 28901353

[ref10] Bica K.; Rogers R. D. Confused Ionic Liquid Ions—a “Liquification” and Dosage Strategy for Pharmaceutically Active Salts. Chem. Commun. 2010, 46 (8), 1215–1217. 10.1039/b925147b. 20449254

[ref11] Martins M. A. R.; Carvalho P. J.; Santos L. M. N. B. F.; Pinho S. P.; Coutinho J. A. P. The Impact of Oligomeric Anions on the Speciation of Protic Ionic Liquids. Fluid Phase Equilib. 2021, 531, 112919 10.1016/j.fluid.2020.112919.

[ref12] Clare Speakman J. Acid Salts of Carboxylic Acids, Crystals with Some ‘very Short” Hydrogen Bonds. In Struct. Bonding (Berlin); Springer Berlin Heidelberg: Berlin, Heidelberg, 1972; pp 141–199.

[ref13] Johansson K. M.; Izgorodina E. I.; Forsyth M.; MacFarlane D. R.; Seddon K. R. Protic Ionic Liquids Based on the Dimeric and Oligomeric Anions: [(AcO)_x_H_x-1_]-. Phys. Chem. Chem. Phys. 2008, 10, 2972–2978. 10.1039/b801405a. 18473045

[ref14] Middendorf M.; Schönhoff M. Nonstoichiometric Protic Ionic Liquids: The Role of Excess Acid in Charge Transport Mechanisms. J. Phys. Chem. B 2024, 128 (12), 2939–2947. 10.1021/acs.jpcb.3c08156. 38484313

[ref15] Ribeiro F. M. S.; Lima C. F. R. A. C.; Silva A. M. S.; Santos L. M. N. B. F. Experimental Evidence for Azeotrope Formation from Protic Ionic Liquids. ChemPhysChem 2018, 19 (18), 2364–2369. 10.1002/cphc.201800335. 29799151

[ref16] Berton P.; Kelley S. P.; Wang H.; Rogers R. D. Elucidating the Triethylammonium Acetate System: Is It Molecular or Is It Ionic?. J. Mol. Liq. 2018, 269, 126–131. 10.1016/j.molliq.2018.08.006.

[ref17] Watanabe H.; Arai N.; Jihae H.; Kawana Y.; Umebayashi Y. Ionic Conduction within Non-Stoichiometric N-Methylimidazole-Acetic Acid Pseudo-Protic Ionic Liquid Mixtures. J. Mol. Liq. 2022, 352, 118705 10.1016/j.molliq.2022.118705.

[ref18] Watanabe H.; Doi H.; Saito S.; Matsugami M.; Fujii K.; Kanzaki R.; Kameda Y.; Umebayashi Y. Hydrogen Bond in Imidazolium Based Protic and Aprotic Ionic Liquids. J. Mol. Liq. 2016, 217, 35–42. 10.1016/j.molliq.2015.08.005.

[ref19] Kohler F.; Liebermann E.; Miksch G.; Kainz C. Thermodynamics of the Acetic Acid-Triethylamine System. J. Phys. Chem. 1972, 76 (19), 2764–2768. 10.1021/j100663a025.

[ref20] Kohler F.; Atrops H.; Kalali H.; Liebermann E.; Wilhelm E.; Ratkovics F.; Salamon T. Molecular Interactions in Mixtures of Carboxylic Acids with Amines. 1. Melting Curves and Viscosities. J. Phys. Chem. 1981, 85 (17), 2520–2524. 10.1021/j150617a021.

[ref21] Kohler F.; Gopal R.; Goetze G.; Atrops H.; Demeriz M. A.; Liebermann E.; Wilhelm E.; Ratkovics F.; Palagyi B. Molecular Interactions in Mixtures of Carboxylic Acids with Amines. 2. Volumetric, Conductimetric, and NMR Properties. J. Phys. Chem. 1981, 85 (17), 2524–2529. 10.1021/j150617a022.

[ref22] Kobrak Mark N.; Nykypanchuk D.; Janssen C. H. C Protic Amine/Acid Mixtures as Solvents for the Extraction of Aqueous Zinc Salts: A Mechanistic Study. J. Mol. Liq. 2023, 380, 121662 10.1016/j.molliq.2023.121662.

[ref23] Kobrak M. N.; Nykypanchuk D.; Janssen C. H. C. Relationship between Liquid Nanoscale Structure in Solvents and the Strength of the Hofmeister Effect in Extraction Experiments. Phys. Chem. Chem. Phys. 2021, 23 (10), 6266–6277. 10.1039/D0CP05973K. 33735349

[ref24] Janssen C. H. C.; Macías-Ruvalcaba N. A.; Aguilar-Martínez M.; Kobrak M. N. Copper Extraction Using Protic Ionic Liquids: Evidence of the Hofmeister Effect. Sep Purif Technol. 2016, 168, 275–283. 10.1016/j.seppur.2016.05.031.

[ref25] Matsumoto M.; Yamaguchi T.; Tahara Y. Extraction of Rare Earth Metal Ions with an Undiluted Hydrophobic Pseudoprotic Ionic Liquid. Metals (Basel) 2020, 10, 502 10.3390/met10040502.

[ref26] Castillo-Ramírez C.; Janssen C. H. C. Pseudo-Protic Ionic Liquids for the Extraction of Metals Relevant for Urban Mining. Ind. Eng. Chem. Res. 2023, 62 (1), 627–636. 10.1021/acs.iecr.2c03159.

[ref27] Alguacil F. J.; Robla J. I. On the Use of Pseudo-Protic Ionic Liquids to Extract Gold(III) from HCl Solutions. Int. J. Mol. Sci. 2023, 24 (7), 6305 10.3390/ijms24076305. 37047277 PMC10094515

[ref28] Padinhattath S. P.; Govindaraj J.; Gardas R. L. Exploring Non-Stoichiometric Pseudoprotic Ionic Liquid for Effective Elimination of Cationic Dyes from Textile Effluent: A Circular Approach. Journal of Water Process Engineering 2024, 58, 104921 10.1016/j.jwpe.2024.104921.

[ref29] Patsos N.; Lewis K.; Picchioni F.; Kobrak M. N. Extraction of Acids and Bases from Aqueous Phase to a Pseudoprotic Ionic Liquid. Molecules 2019, 24 (5), 894 10.3390/molecules24050894. 30836603 PMC6429149

[ref30] Kobrak M. N.; Yager K. G. X-Ray Scattering and Physicochemical Studies of Trialkylamine/Carboxylic Acid Mixtures: Nanoscale Structure in Pseudoprotic Ionic Liquids and Related Solutions. Phys. Chem. Chem. Phys. 2018, 20, 18539–18646. 10.1039/C8CP02854K. 29955736

[ref31] Kobrak M.; Nykypanchuk D. Small Angle X-Ray Studies of Short-Range Order in Non-Stoichiometric Pseudoprotic Ionic Liquids: The Influence of Chemical Structure. Phys. Chem. Chem. Phys. 2023, 25, 26049–26059. 10.1039/D3CP01853A. 37727108

[ref32] Lajovic A.; Tomšič M.; Jamnik A. Structural Study of Simple Organic Acids by Small-Angle X-Ray Scattering and Monte Carlo Simulations. Acta Chim. Slov. 2012, 59 (3), 520–527. 24061305

[ref33] Noël J. A.; Leblanc L. M.; Patterson D. S.; Kreplak L.; Fleischauer M. D.; Johnson E. R.; White M. A. Clusters in Liquid Fatty Acids: Structure and Role in Nucleation. J. Phys. Chem. B 2019, 123, 7043–7054. 10.1021/acs.jpcb.9b05017. 31322886

[ref34] Odinokov S. E.; Glazunov V. P.; Nabiullin A. A. Infrared Spectroscopic Studies of Hydrogen Bonding in Triethylammonium Salts. Part 3.—Strong Hydrogen Bonding. J. Chem. Soc., Faraday Trans. 2 1984, 80 (8), 899–908. 10.1039/F29848000899.

[ref35] Quilès F.; Burneau A. Infrared and Raman Spectra of Alkaline-Earth and Copper(II) Acetates in Aqueous Solutions. Vib Spectrosc 1998, 16 (2), 105–117. 10.1016/S0924-2031(98)00004-6.

[ref36] Odinokov S. E.; Iogansen A. V. Torsional γ(OH) Vibrations, Fermi Resonance [2γ(OH) ⇐ ν(OH)] and Isotopic Effects in I. R. Spectra of H-Complexes of Carboxylic Acids with Strong Bases. Spectrochim Acta A 1972, 28 (12), 2343–2350. 10.1016/0584-8539(72)80214-9.

[ref37] Odinokov S. E.; Nabiullin A. A.; Mashkovsky A. A.; Glazunov V. P. Effects of Isotopic Substitution on Spectral Parameters of the ν_AH_ and v_B+H_ Infrared Bands in Molecular and Ionic H-Complexes. Spectrochim Acta A 1983, 39 (12), 1055–1063. 10.1016/0584-8539(83)80125-1.

[ref38] Van Hoozen B. L. Jr; Petersen P. B. Vibrational Tug-of-War: The PKA Dependence of the Broad Vibrational Features of Strongly Hydrogen-Bonded Carboxylic Acids. J. Chem. Phys. 2018, 148 (13), 134309 10.1063/1.5026675. 29626887

[ref39] Nibbering E. T. J.; Elsaesser T. Ultrafast Vibrational Dynamics of Hydrogen Bonds in the Condensed Phase. Chem. Rev. 2004, 104 (4), 1887–1914. 10.1021/cr020694p. 15080715

[ref40] Brubach J.-B.; Mermet A.; Filabozzi A.; Gerschel A.; Lairez D.; Krafft M. P.; Roy P. Dependence of Water Dynamics upon Confinement Size. J. Phys. Chem. B 2001, 105 (2), 430–435. 10.1021/jp002983s.

[ref41] Fameau A.-L.; Arnould A.; Saint-Jalmes A. Responsive Self-Assemblies Based on Fatty Acids. Curr. Opin. Colloid Interface Sci. 2014, 19 (5), 471–479. 10.1016/j.cocis.2014.08.005.

[ref42] Mondal M.; Malik S.; De S.; Bhattacharyya S.; Saha B. Employment and Resurrection of Surfactants in Bipyridine Promoted Oxidation of Butanal Using Bivalent Copper at NTP. Res. Chem. Intermed. 2017, 43, 1651 10.1007/s11164-016-2721-6.

[ref43] Coeck R.; De Vos D. E. One-Pot Reductive Amination of Carboxylic Acids: A Sustainable Method for Primary Amine Synthesis. Green Chem. 2020, 22 (15), 5105–5114. 10.1039/D0GC01441A.

[ref44] Barratt M. D. Quantitative Structure-Activity Relationships (QSARs) for Skin Corrosivity of Organic Acids, Bases and Phenols: Principal Components and Neural Network Analysis of Extended Datasets. Toxicology in Vitro 1996, 10 (1), 85–94. 10.1016/0887-2333(95)00101-8. 20650186

[ref45] Cistola D. P.; Small D. M.; Hamilton J. A. Ionization Behavior of Aqueous Short-Chain Carboxylic Acids: A Carbon-13 NMR Study. J. Lipid Res. 1982, 23 (5), 795–799. 10.1016/S0022-2275(20)38114-1. 7119577

[ref46] Saika A.; Gutowsky H. S Dissociation, Chemical Exchange, and the Proton Magnetic Resonance in Some Aqueous Electrolytes. J. Chem. Phys. 1953, 21 (10), 1688–1694. 10.1063/1.1698644.

[ref47] Brabson G. D. Nmr Analysis of Water-Acetic Acid Solutions. J. Chem. Educ. 1969, 46 (11), 754 10.1021/ed046p754.

[ref48] Vogel M. NMR Studies on Simple Liquids in Confinement. Eur. Phys. J. Spec Top 2010, 189 (1), 47–64. 10.1140/epjst/e2010-01309-9.

[ref49] Koeppe B.; Pylaeva S. A.; Allolio C.; Sebastiani D.; Nibbering E. T. J.; Denisov G. S.; Limbach H.-H.; Tolstoy P. M. Polar Solvent Fluctuations Drive Proton Transfer in Hydrogen Bonded Complexes of Carboxylic Acid with Pyridines: NMR, IR and Ab Initio MD Study. Phys. Chem. Chem. Phys. 2017, 19 (2), 1010–1028. 10.1039/C6CP06677A. 27942642

[ref50] Sharif S.; Denisov G. S.; Toney M. D.; Limbach H.-H. NMR Studies of Coupled Low- and High-Barrier Hydrogen Bonds in Pyridoxal-5‘-Phosphate Model Systems in Polar Solution. J. Am. Chem. Soc. 2007, 129 (19), 6313–6327. 10.1021/ja070296+. 17455937

[ref51] Wylie L.; Kéri M.; Udvardy A.; Hollóczki O.; Kirchner B. On the Rich Chemistry of Pseudo-Protic Ionic Liquid Electrolytes. ChemSusChem 2023, 16 (20), e202300535 10.1002/cssc.202300535. 37364035

[ref52] Jacobi R.; Joerg F.; Steinhauser O.; Schröder C. Emulating Proton Transfer Reactions in the Pseudo-Protic Ionic Liquid 1-Methylimidazolium Acetate. Phys. Chem. Chem. Phys. 2022, 24 (16), 9277–9285. 10.1039/D2CP00643J. 35403653 PMC9020328

[ref53] Jones M. N.; Chapman D. Micelles, Monolayers and Biomembranes; Wiley-Liss: New York, 1995.

[ref54] Salentinig S.; Phan S.; Darwish T. A.; Kirby N.; Boyd B. J.; Gilbert E. P. PH-Responsive Micelles Based on Caprylic Acid. Langmuir 2014, 30 (25), 7296–7303. 10.1021/la500835e. 24905895

[ref55] Kaibara K.; Iwata E.; Eguchi Y.; Suzuki M.; Maeda H. Dispersion Behavior of Oleic Acid in Aqueous Media: From Micelles to Emulsions. Colloid Polym. Sci. 1997, 275 (8), 777–783. 10.1007/s003960050147.

[ref56] Agmon N. The Grotthuss Mechanism. Chem. Phys. Lett. 1995, 244 (5), 456–462. 10.1016/0009-2614(95)00905-J.

[ref57] Zhang J.; Yao J.; Li H. Solvent Effect on the Ionicity of Protic Ionic Liquid: 1-Methylimidazolium-Acetic Acid. J. Phys. Chem. B 2022, 126 (11), 2279–2284. 10.1021/acs.jpcb.2c00973. 35271286

[ref58] Sutter J.; Haese C.; Graf R.; Hunger J. Charge Transport in Protic Ionic Liquids: Effect of Protonation State in 1-Methylimidazolium–Acetate/Trifluoroacetate Mixtures. J. Mol. Liq. 2023, 390, 122975 10.1016/j.molliq.2023.122975.

[ref59] Joerg F.; Sutter J.; van Dam L.; Kanellopoulos K.; Hunger J.; Schröder C. Comparative Analysis of Dielectric Spectra in Protic Ionic Liquids: Experimental Findings and Computational Molecular Decomposition. J. Mol. Liq. 2024, 396, 123834 10.1016/j.molliq.2023.123834.

[ref60] Yaghini N.; Gómez-González V.; Varela L. M.; Martinelli A. Structural Origin of Proton Mobility in a Protic Ionic Liquid/Imidazole Mixture: Insights from Computational and Experimental Results. Phys. Chem. Chem. Phys. 2016, 18 (33), 23195–23206. 10.1039/C6CP03058K. 27499376

