# Supplementary material for: Proton Transfer Equilibrium in Pseudoprotic Ionic Liquids: Inferences on Ionic Populations
Source: J Phys Chem B. 2025 Jan 17;129(4):1376–86. doi: 10.1021/acs.jpcb.4c07150 (PMC11789136; doi:10.1021/acs.jpcb.4c07150)

SUPPORTING INFORMATION:
Proton Transfer in Pseudoprotic Ionic Liquids: Inferences on Ionic Populations

Mark N.Kobrak[a,b *] , Dmytro Nykypanchuk[c], Ankit Jain[a,b,d], Eddie Louz[a] and Andrzej A. Jarzecki[a,b]

[a] Department of Chemistry and Biochemistry, Brooklyn College of the City University of New York, 2900 Bedford Ave., Brooklyn, NY 11210;
[b] Department of Chemistry, The Graduate Center of the City University of New York, 365 Fifth Ave., New York, NY, 10016;
[c]Center for Functional Nanomaterials, Brookhaven National Laboratory, Upton, NY 11973;
[d] Department of Biochemistry, The Graduate Center of the City University of New York, 365 Fifth Ave., New York, NY, 10016

Section S1: Numerical fitting for SAXS data for H_2O/HBA χ=0.2, 0.3

To gain additional insight on the low q structure of the H_2O/HBA system, we perform a simple numerical analysis of the SAXS profiles for the χ=0.2 and 0.3 mixtures. The analysis consists of a numerical fit of the data to four Gaussian functions and a linear baseline.

$$S(q) = a + bq + \sum_{i=1}^{4} A_i \exp\left[-\frac{1}{2}\frac{(q-q_{0i})^2}{w_i^2}\right] \quad \text{(S1)}$$

The wavenumber q was expressed in units of $Å^{-1}$. For each dataset, the function was fit using Mathematica 13.0 to implement the above function.

This is intended to provide a qualitative guide to the structure at low q. A rigorous analysis would require a more detailed structural model for the system, which is not available at this time.

For each of the two cases fit, a plot of the total fitted function and its components in Equation S1 is given, along with the residuals and the values of the fitted parameters. In each case, the local structure peak is given as i=1 (i.e. the peak center is q_{01}). The χ=0.3 sample shows a second, much smaller peak near the same position, but we consider only the primary peak. The peak centers are given in Table S.1.

Table S.1: Peak centers and corresponding lengthscales for the H_2O/HBA χ=0.2, 0.3 samples.

χ	q($Å^{-1}$)	D (Å)
0.2	0.302	20.8
0.3	0.392	16.0

χ=0.2

Figure S.1: Left: SAXS data (red, 1/5) and fitting functions (blue) for H_2O/HBA χ=0.2. Right: Fit residuals.

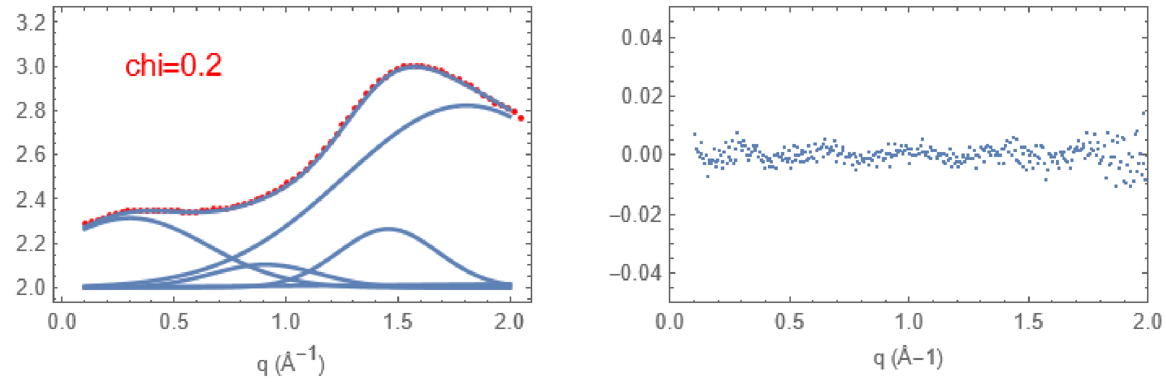

Table S.2: Fitting parameters for H_2O/HBA, χ=0.2. q is in units of Å^{-1}.

χ	a	b	q01	w1	A1	q02	w2	A2	q03	w3	A3	q04	w4	A4
0.2	2.00	0.00747	0.303	0.347	0.315	1.81	0.548	0.824	1.46	0.222	0.265	0.908	0.237	0.104

χ=0.3

Figure S.2: Left: SAXS data (red, 1/5) and fitting functions (blue) for H_2O/HBA χ=0.3. Right: Fit residuals.

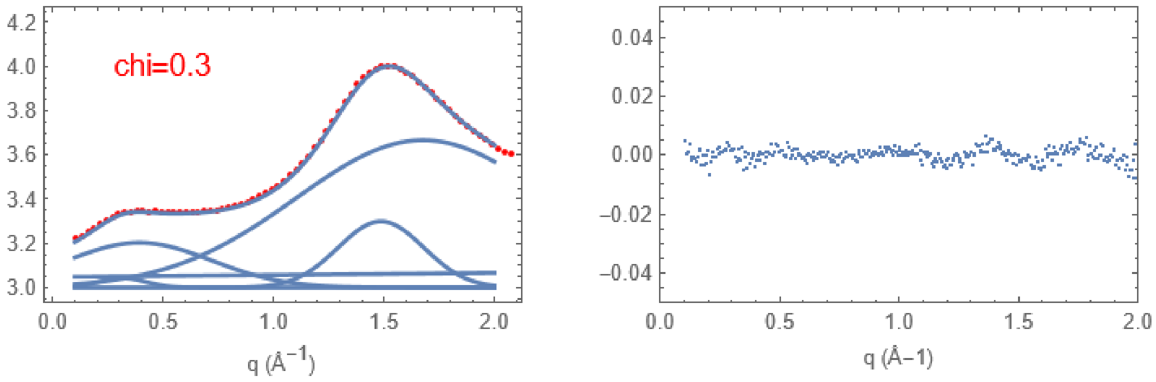

Table S.3: Fitting parameters for H_2O/HBA, χ=0.3.

χ	a	b	q01	w1	A1	q02	w2	A2	q03	w3	A3	q04	w4	A4
0.3	3.05	0.00937	0.392	0.327	0.203	1.68	0.575	0.667	1.49	0.193	0.300	0.315	0.107	0.0431

Section S2: Infrared Spectra

Figure S.3: Infrared spectra for trihexylamine/butyric acid (left) and water/butyric acid (right).

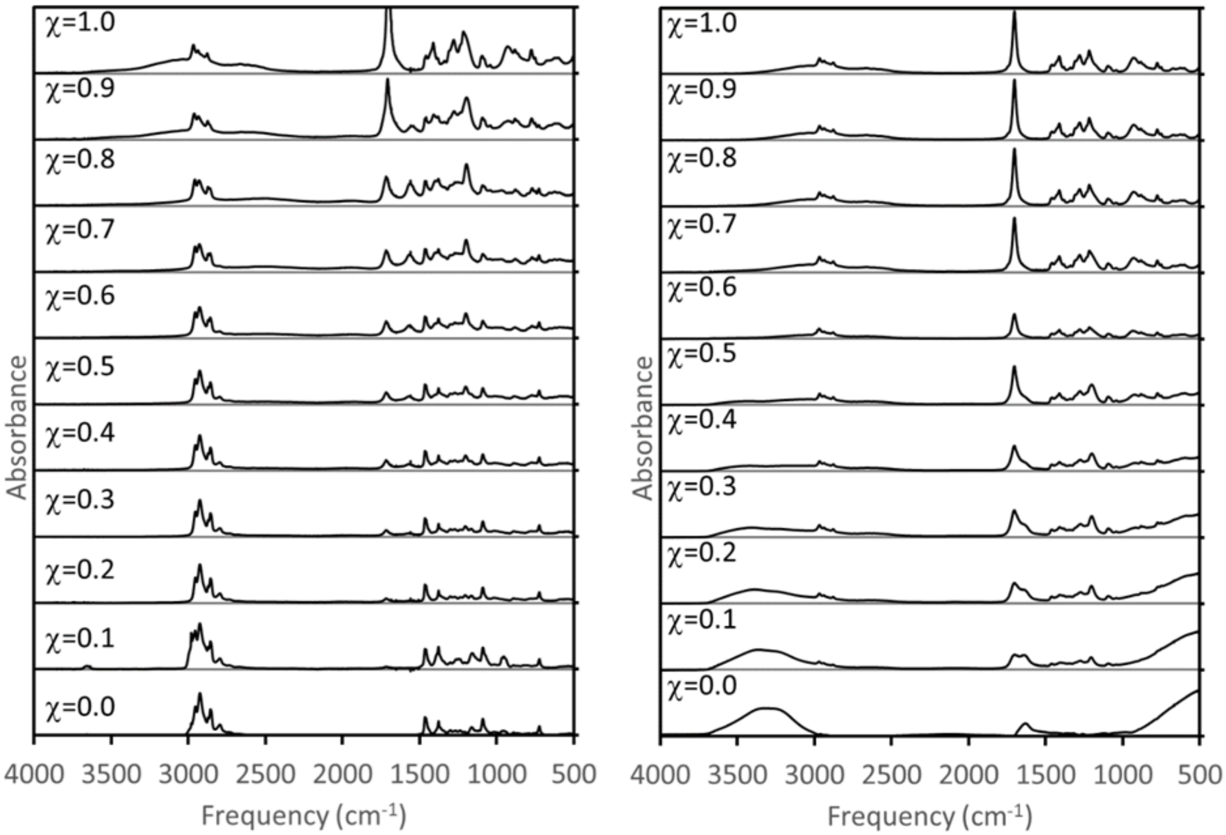

Section S3: Fitting Infrared Spectral Data

Data were fitted using Mathematica 13.3.

As described in the text of Section III.B, the first step is to fit the spectrum of pure butyric acid from 3000 cm^{-1} to 4000 cm^{-1} according to the formula

$$f(\nu) = a_0 + A_0 e^{-\frac{1}{w_0^2}(\nu - \nu_{00})^2} \qquad \text{(S2)}$$

Fitting parameters for this form are given in Table S.4, and the fitted function is shown in Figure S1. These parameters are used in fitting the χ=0.3 data, with only the value of A_0 allowed to change.

Table S.4: Fitting parameters for pure butyric acid over the range 3000 cm^{-1} to 4000 cm^{-1}.

a_0	A_0	w_o (cm^{-1})	ν_{00} (cm^{-1})
0.005554	0.1041	203.4	2995

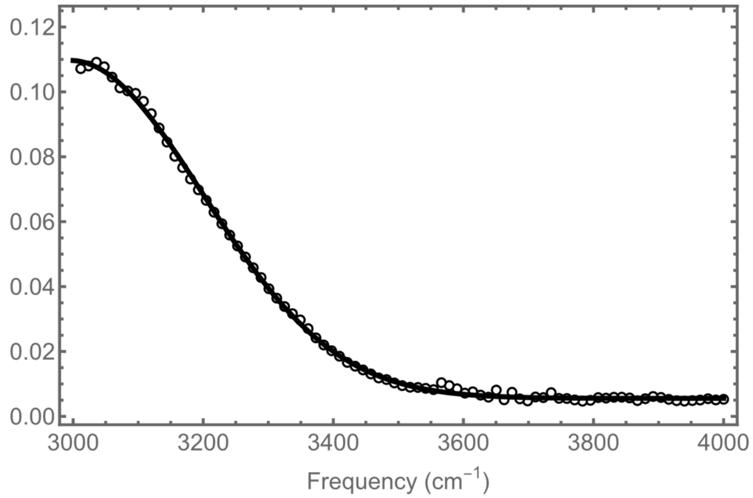

Figure S.4: Data (open circles, 1/50 shown) and Gaussian fit (solid line) for the infrared spectrum of pure butyric acid.

The function for fitting the χ=0.3 data is

$$f(\nu) = a_1 + A_0 e^{-\frac{1}{w_0^2}(\nu - \nu_{00})^2} + \sum_{i=1}^{3} A_i e^{-\frac{1}{w_i^2}(\nu - \nu_{0i})^2} \qquad \text{(S3)}$$

The fitted function is shown in Figure S.4, along with the residuals:

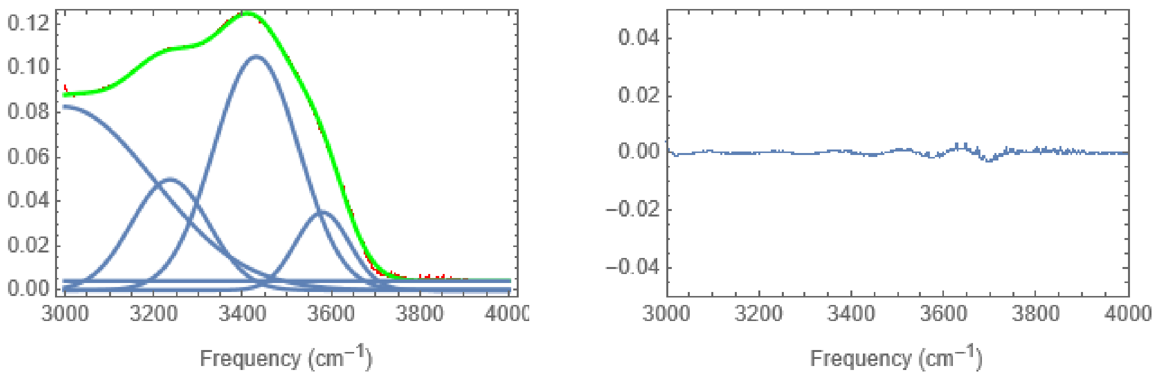

Figure S.5: Left: Data, fitted Function(green), and components of the fitting function (blue). Right: Residuals.

Table S.5: Fitting parameters for χ=0.3 over the range 3000 cm^{-1} to 4000 cm^{-1}.

i	A_i	w_i (cm^{-1})	ν_{0i} (cm^{-1})
1	0.1054	96.13	3431
2	0.03512	61.13	3580.
3	0.04988	86.90	3236
a_1=0.004167	A_0=0.08283		

Figure S.6 displays the final fit in comparison to the experimental data, and includes the overlap with the fitted acid spectrum.

Figure S.6. Fitting of the water spectrum. Open circles: Experimental data (1/5 pts). Black: Gaussian component terms in the fit. Gray-Dashed: Fitted shoulder for butyric acid. Gray-Solid: Sum over all components.

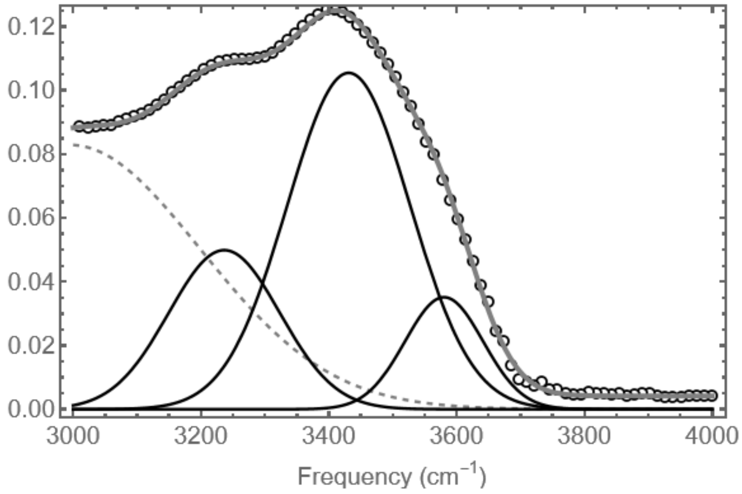

Section S4: NMR Spectra of NPPIL Mixtures

^{1}H- and ^{13}C-NMR spectra for all compositions of the mixtures studied in this work are given below.

^{1}H-NMR Spectroscopy: The proton ^{1}H-NMR spectra are given in Figures S.6-S.7. The water line at 4.8 ppm is associated with the calibration phase (except in the pure water spectrum), arising from contamination of the heavy water used in dissolution. The intensity of this line varies from spectrum to spectrum owing to water generated in the gradual breakdown of the silyl compound used for calibration, though the chemical shift of the calibration line is not affected.

The chemical shifts for the protons indicated in Figure S.6 are given in Figure 7 in the main body of this work, and their interpretation is discussed in the surrounding text.

There is additional information contained in the labile proton peak widths, but the intensity of the peak is low and attempting an appropriate fit could lead to overinterpretation. We therefore do not pursue this analysis.

Figure S.6. ^{1}H-NMR spectrum of trihexylamine/butyric acid mixtures ranging from pure butyric acid (χ=1.0) to pure trihexylamine (χ=0.0). Note the break in the x-axis and the increased scale at high chemical shifts, necessary to render the labile proton signal visible.

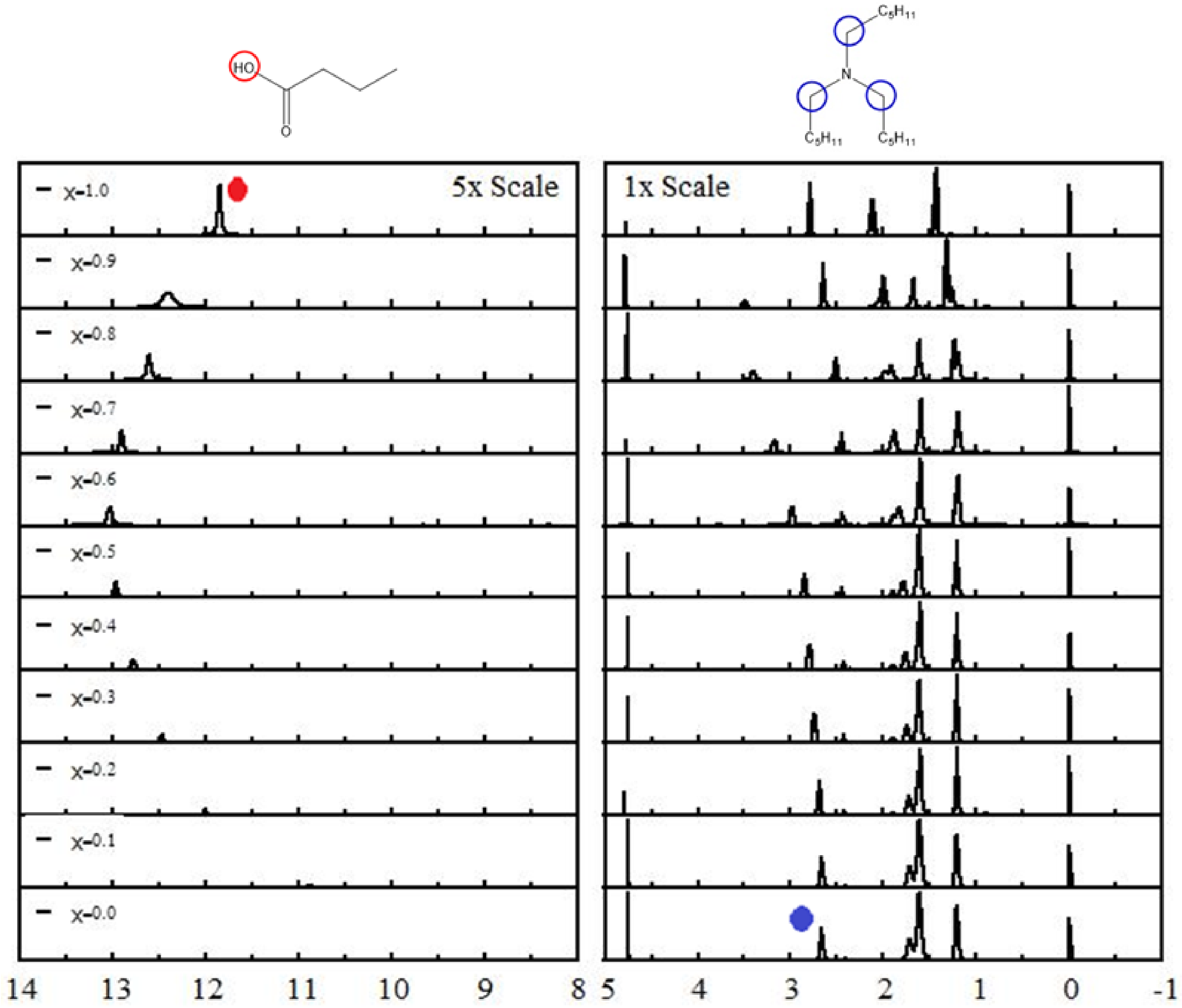

Figure S.7. ^{1}H-NMR spectrum of water/butyric acid mixtures ranging from pure butyric acid (top, χ=1.0) to pure water (χ=0.0).

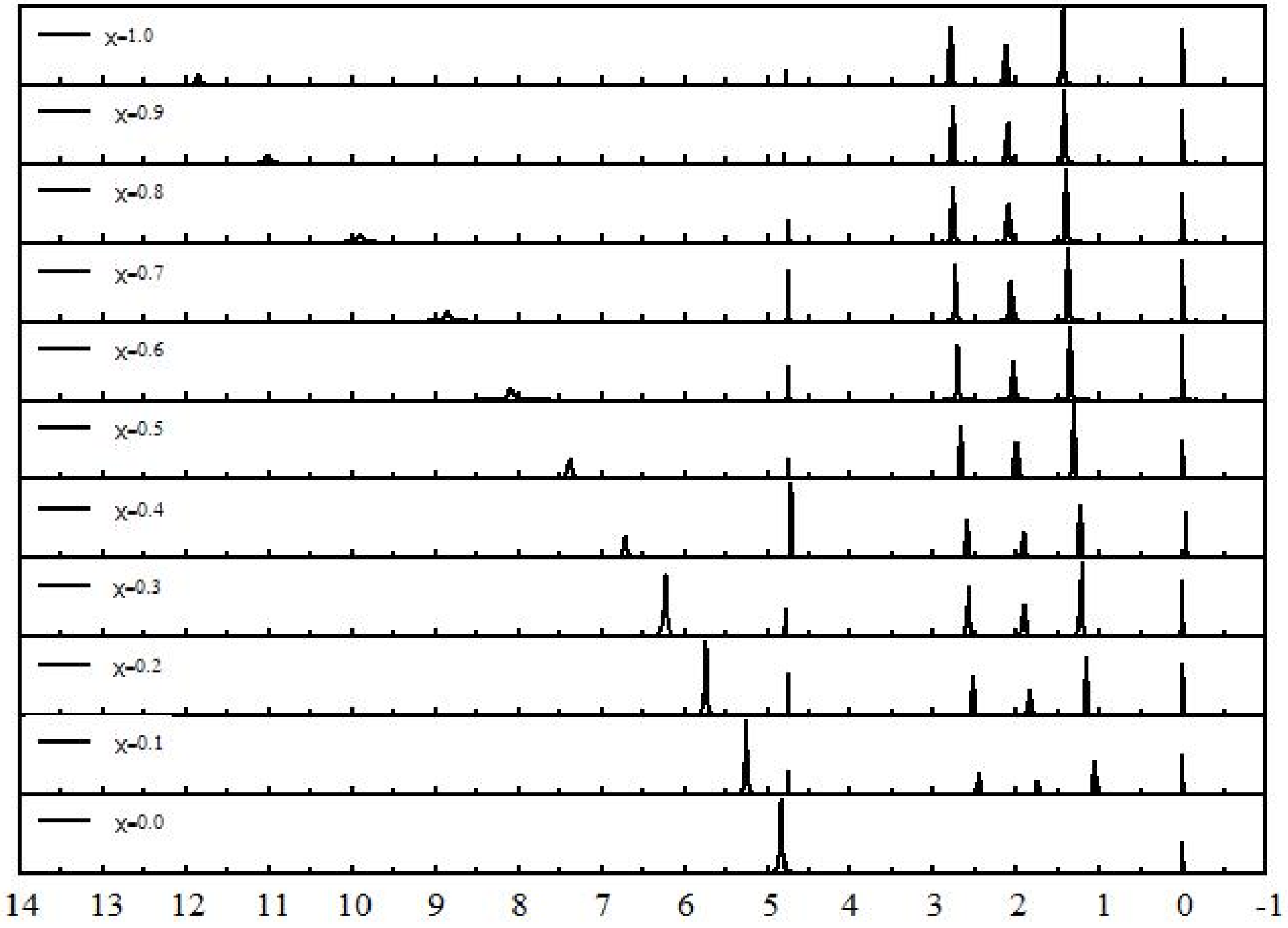

^{13}C-NMR Spectroscopy: The proton ^{13}C-NMR spectra for H_2O/HBA and T6A/HBA are given in Figures S.8-S.9. The chemical shifts for the carboxyl carbon in Figure S.8 are given in Figure 8 of the main body of this work, and their interpretation is discussed in the surrounding text.

Figure S.8. Top: ^{13}C-NMR spectra for the T6A/HBA system. Bottom: As above, with scale increased to aid visualization of the carboxyl carbon.

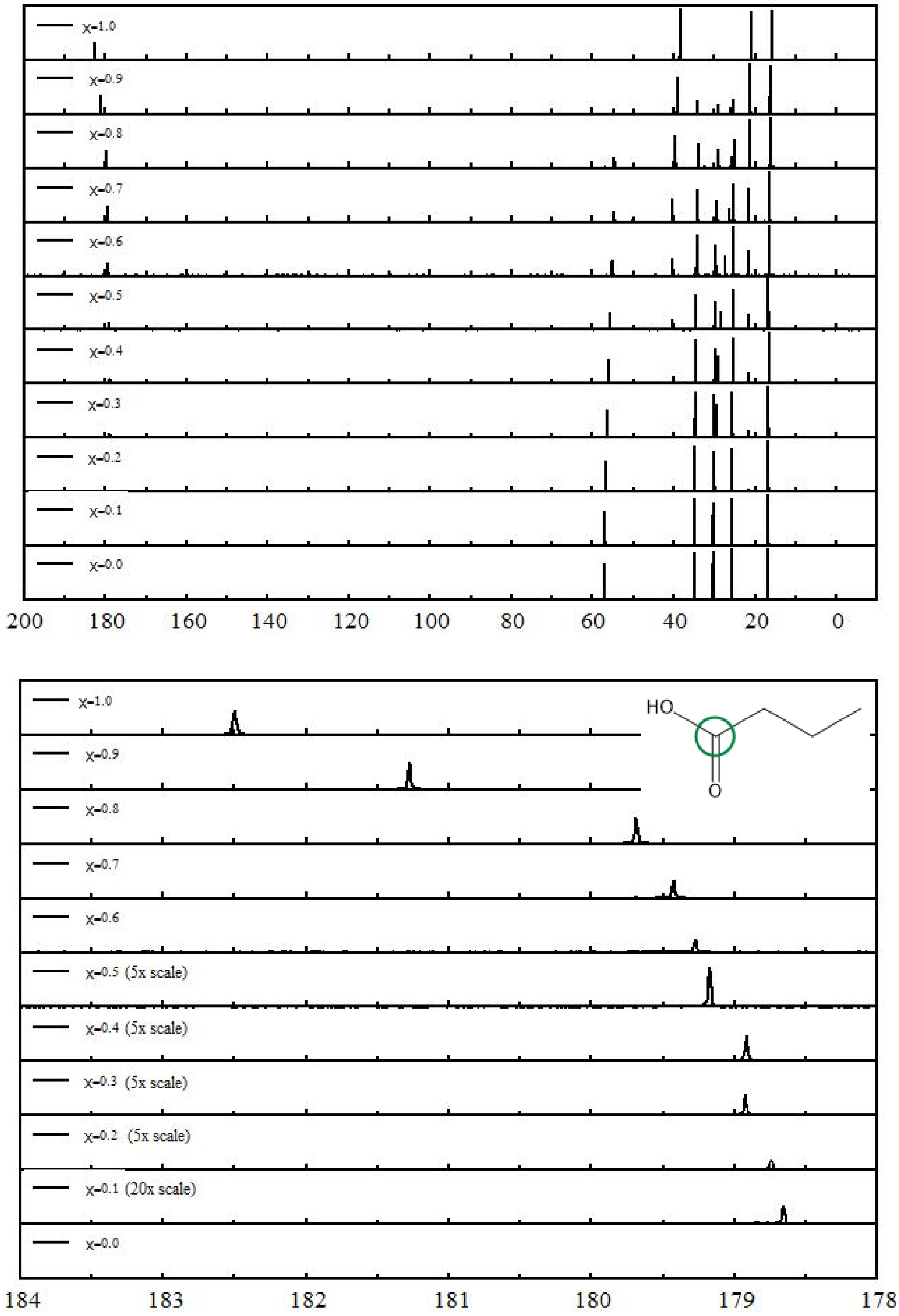

Figure S.9: Top: ^{13}C-NMR spectra for H_2O/HBA system. Bottom: As above, with scale increased to aid visualization of the carboxyl carbon.

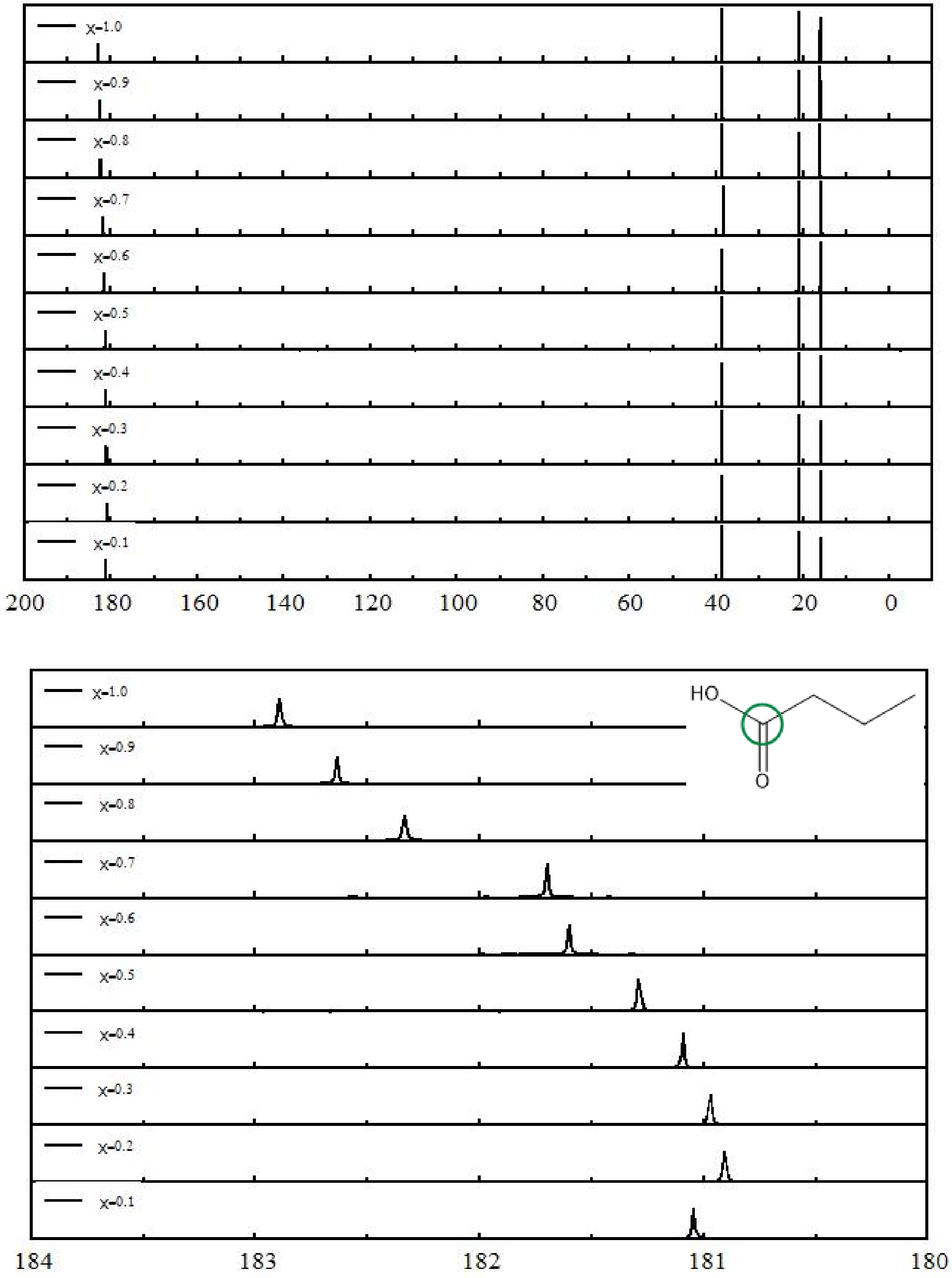

Section S5: Numerical Modeling of NMR Data

Table S.6 gives the values of the reference chemical shifts for the model, and how they are experimentally determined. The pure amine was used to determine the chemical shift of the unprotonated T6A (δ_{nT6A}^{H}), and the chemical shift of the labile proton in protonated T6A (δ_{HT6A}^{H}) was taken from the spectrum of a mixture of 0.9 mole fraction T6A/0.1 mole fraction trichloroacetic acid as described in the previous section. The chemical shifts for the neutral monomer (δ_{HA}^{H} and δ_{HA}^{C}) were calculated from a dilute solution of butyric acid in acetone, and those for the neutral dimer (δ_{H2A2}^{H} and δ_{H2A2}^{C}) were taken from the spectrum of the pure acid (i.e. the butyric acid was assumed to be purely monomeric in dilute solution and purely dimeric in the neat acid).

It was necessary to use the χ=0.9 data for the T6A/HBA mixtures to acquire some of the necessary reference chemical shifts. For this purpose, it was assumed that the amine was fully protonated, which permitted determination of δ_{pT6A}^{H}. A further assumption was that all acid molecules were present either as dimeric neutral or dimeric anionic species, which permitted calculation of δ_{HA2}^{H} and δ_{HA2}^{C}.

Table S.6: Reference states used for chemical shift parameters in Equations 5-7 and 9 (see main body of this work). Spectra for these systems are reported in Sections S4 (NPPILs) and S6 (T6A and HBA in non-NPPIL solvent environments).

State	Chemical Shift	Notes
HA	δ_{HA}^{H}=10.4	Based on dilute butyric acid solution in acetone
	δ_{HA}^{C}=174.4	
A^-	δ_{A}^{C}=184.9	Based on saturated solution of sodium butyrate in methanol
H_2A_2	δ_{H2A2}^{H}=11.8	Based on pure butyric acid, assumed to be 100% dimerized
	δ_{H2A2}^{C} =182.5	
HA_2^-	δ_{HA2}^{H}=20.2	Based on fit at χ=0.9, assumes full ionization of acid into dimer
	δ_{HA2}^{C}=175.7	
HT6A	δ_{HT6A}^{H}=8.8	Based on spectrum of 0.1 mole fraction chloroacetic acid/0.9 mole fraction trihexylamine (no solvent)
pT6A	δ_{pT6A}^{H}=3.5	Assumes full protonation at χ=0.9
nT6A	δ_{nT6A}^{H}=2.7	Based on chemical shift in pure T6A samples

The results of the fitting process are given in Figure S.10. With the exception of the ^{13}C chemical shift for χ=0.1, the results are remarkably good. The deviation at χ=0.1 reflects a contradiction between the different chemical shifts utilized in the analysis. The chemical shift of the nitrogen-proximate -CH_2- group for χ=0.1 is 2.7, the same as for pure trihexylamine and therefore taken as the limit of the chemical shift for the unionized species. This is consistent with the labile proton chemical shift, which can be adequately reproduced through a linear combination of the unionized acid forms. However, the observed chemical shift of the carboxyl carbon is too high to result from the parameters given for the fully unionized species in the model. The inconsistency likely arises from the fact that fitting for the dimeric reference states relies on data from χ=0.9 and 1, which constitutes a very different solvent environment than the nonpolar environment associated with the high T6A concentration at low χ values. Nevertheless, the high quality of the fit for all other points, particularly for χ>0.5, lends credibility to the analysis.

Figure S.10. Experimental (circles) vs. calculated (joined squares) chemical shifts as a function of χ. Top: Carboxyl ^{13}C chemical shift. Middle: Labile proton ^{1}H chemical shift. Bottom: N-proximate ^{1}H -CH_2- chemical shift.

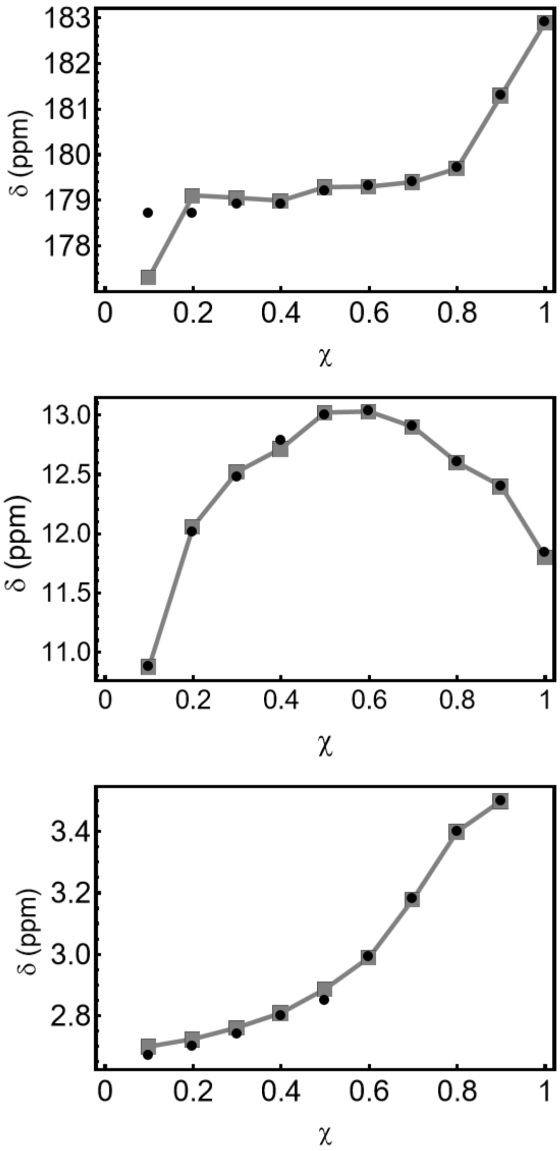

Section S6: NMR Reference States

Spectra for the reference states described in Table S.6 are given below.

^{1}H-NMR Spectrum of 0.01M Butyric Acid in Acetone-d6

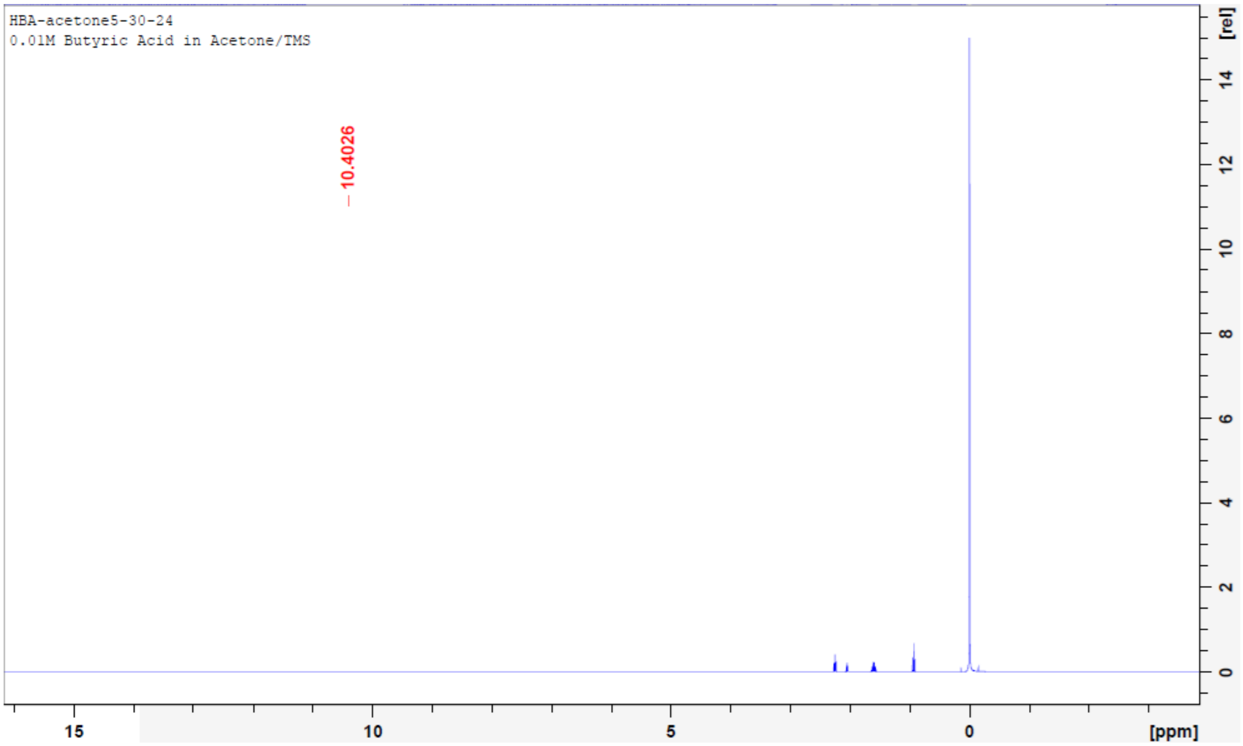

^{1}H-NMR Spectrum of 0.01M Butyric Acid in Acetone-d6, rescaled

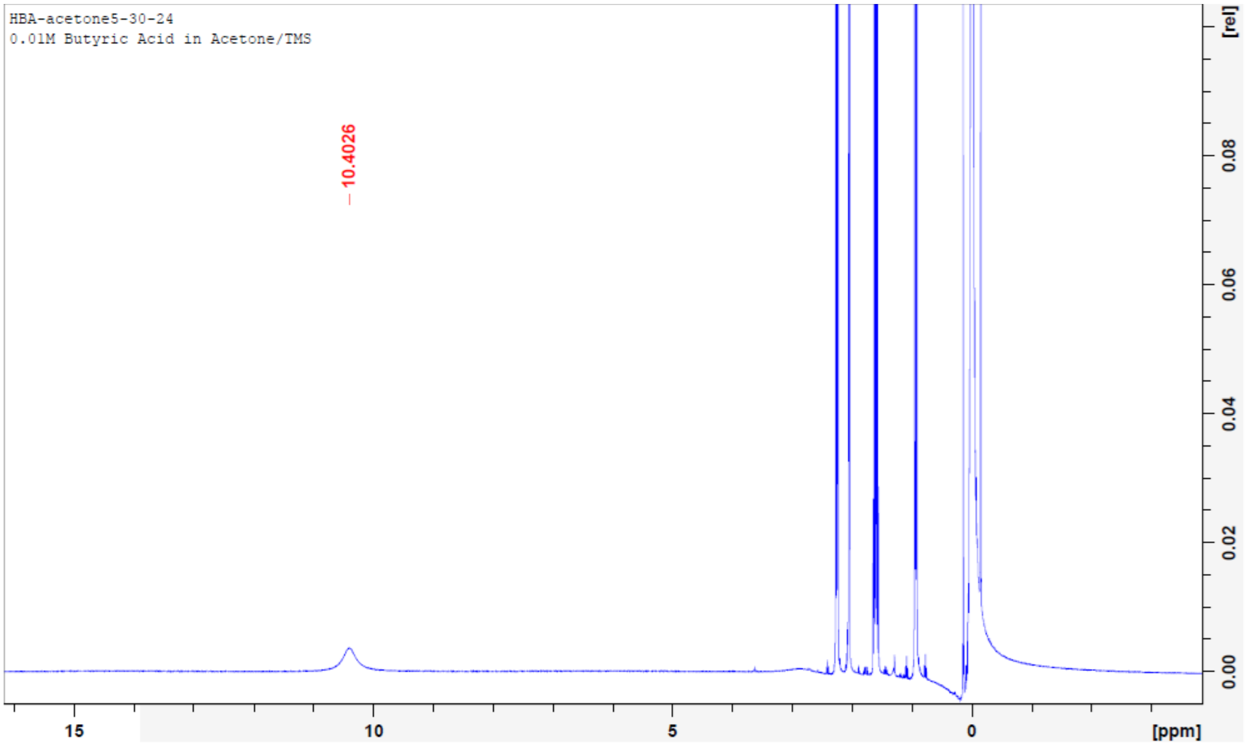

^{13}C-NMR Spectrum of 0.01M Butyric Acid in Acetone-d6

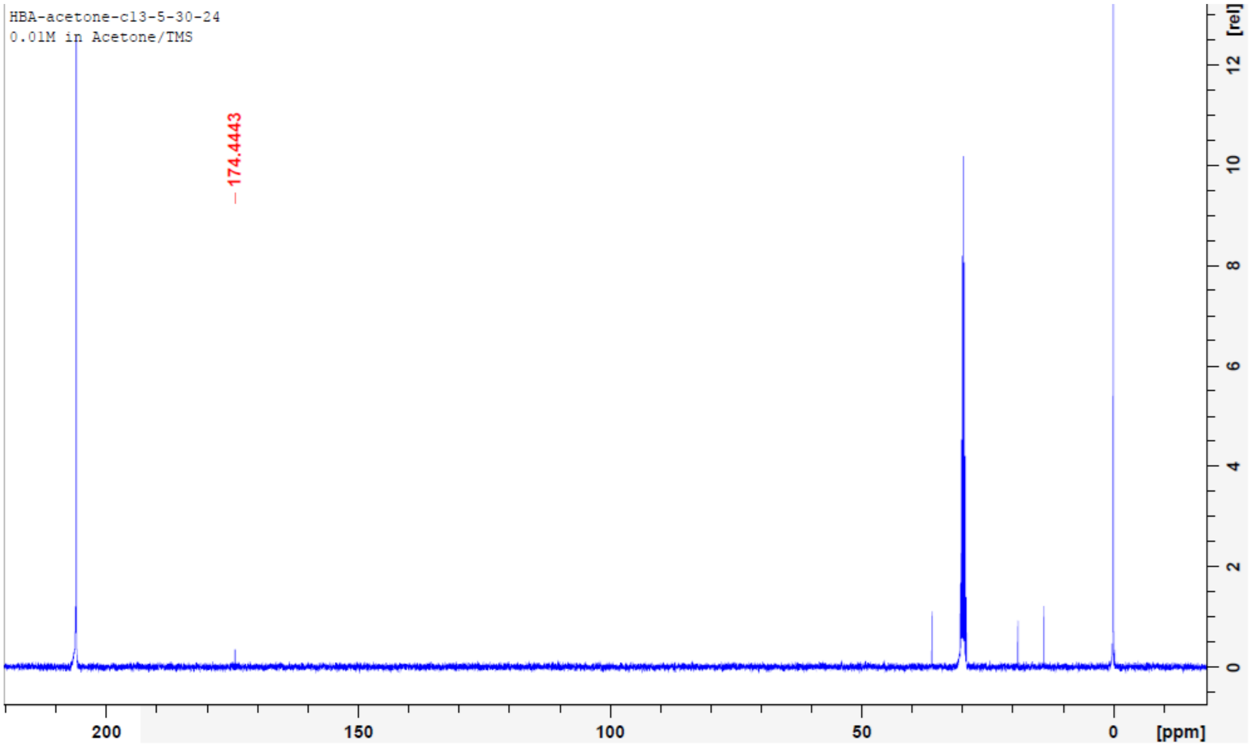

^{13}C-NMR Spectrum of Saturated Solution of Sodium Butyrate in Methanol

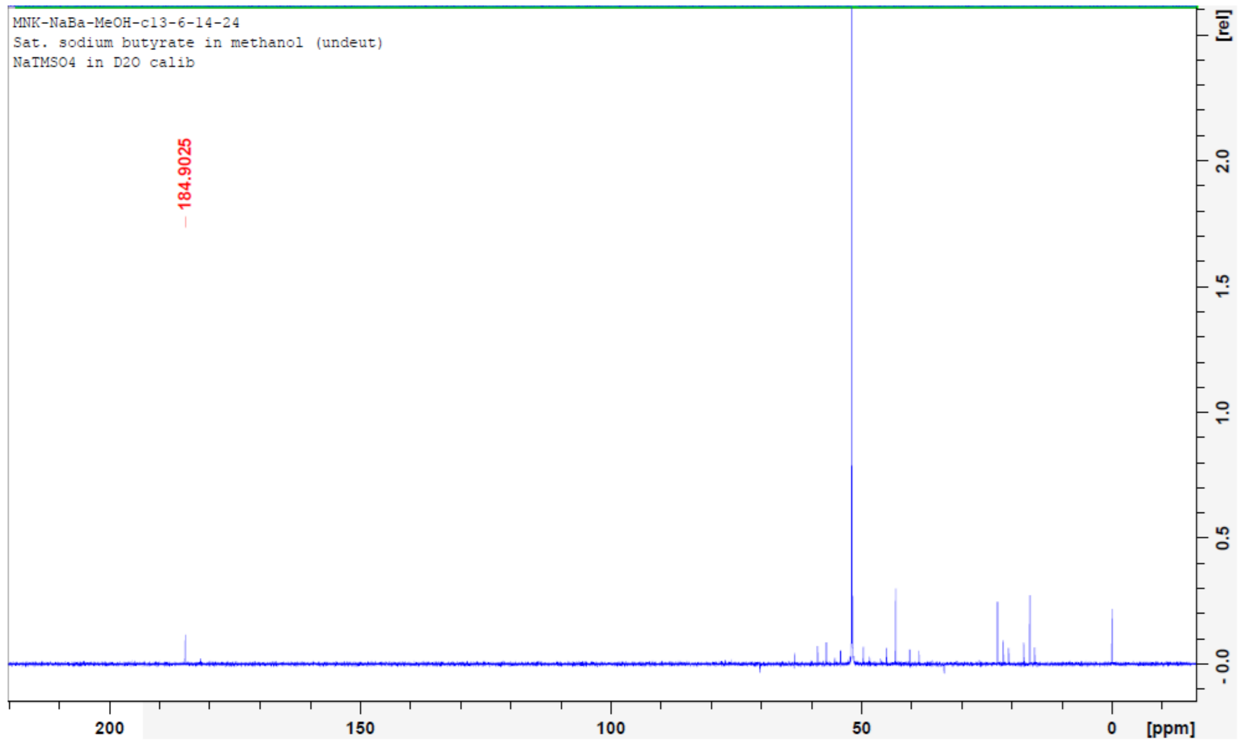

^{1}H-NMR Spectrum of Mixture: 0.1 Mole Fraction Trichloroacetic Acid/0.9 Mole Fraction Trihexylamine

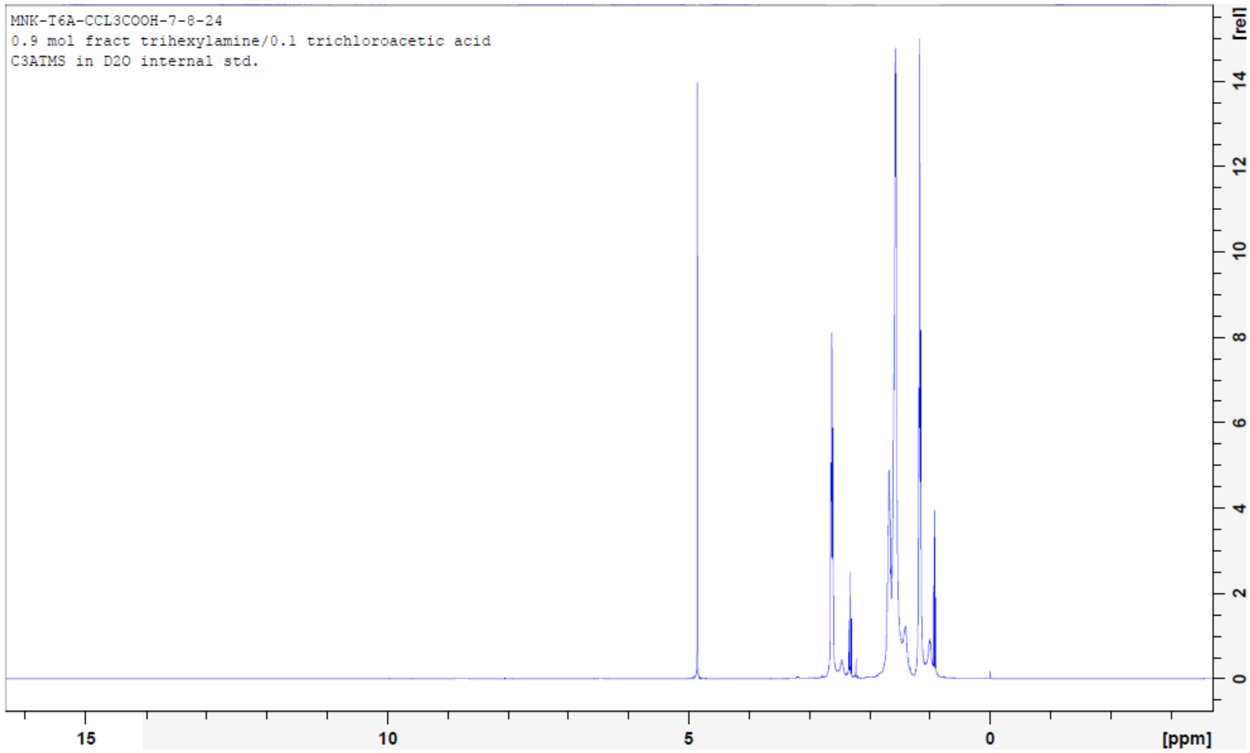

^{1}H-NMR Spectrum of Mixture: 0.1 Mole Fraction Trichloroacetic Acid/0.9 Mole Fraction Trihexylamine, Rescaled

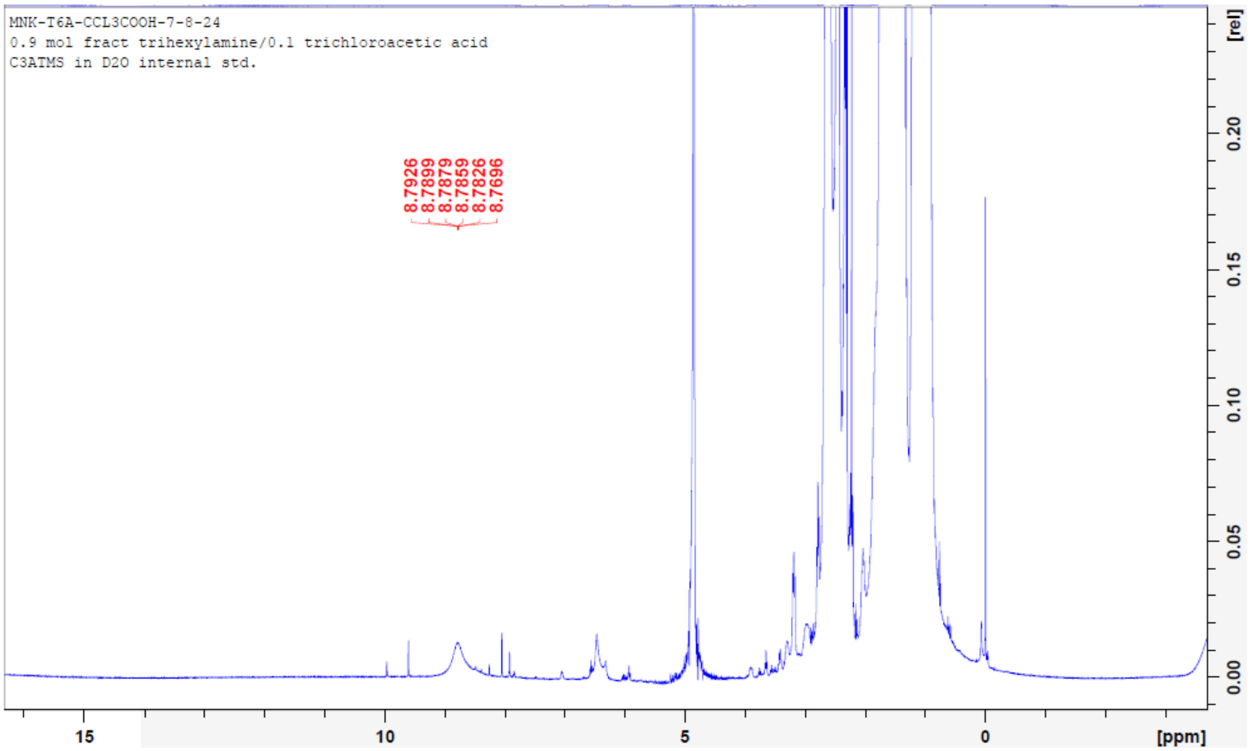

Supplement: Supplementary file 1 — jp4c07150_si_001.pdf [file jp4c07150_si_001.pdf]
